# A qualitative study of imaginary pills and open-label placebos in test anxiety

**DOI:** 10.1371/journal.pone.0291004

**Published:** 2023-09-01

**Authors:** Sarah Buergler, Dilan Sezer, Alexander Busch, Marlon Enzmann, Berfin Bakis, Cosima Locher, Niels Bagge, Irving Kirsch, Claudia Carvalho, Jens Gaab

**Affiliations:** 1 Division of Clinical Psychology and Psychotherapy, Faculty of Psychology, University of Basel, Basel, Switzerland; 2 Department of Consultation-Liaison Psychiatry and Psychosomatic Medicine, University Hospital Zurich, University of Zurich, Zurich, Switzerland; 3 Institute for Emotion-Focused Therapy, Roskilde, Denmark; 4 Program in Placebo Studies, Beth Israel Deaconess Medical Center and Harvard Medical School, Boston, MA, United States of America; 5 Department of Clinical and Health Psychology, Instituto Superior de Psicologia Aplicada (ISPA), Lisbon, Portugal; MSH Medical School Hamburg, GERMANY

## Abstract

**Background:**

The efficacy of open-label placebos (OLPs) has been increasingly demonstrated and their use holds promise for applications compatible with basic ethical principles. Taking this concept one step further an imaginary pill (IP) intervention without the use of a physical pill was developed and tested in a randomized controlled trial (RCT). To explore participants’ experiences and views, we conducted the first qualitative study in the field of IPs.

**Methods:**

A reflexive thematic analysis (RTA) of semi-structured interviews with test anxious students (*N* = 20) was nested in an RCT investigating an IP and OLP intervention. In addition, open-ended questions from the RCT were evaluated (*N* = 114) to corroborate the RTA and pill characteristics were included to more accurately capture the IP experience.

**Results:**

Four key themes were identified: (1) attitude towards the intervention, (2) applicability of the intervention, (3) experience of effects, and (4) characteristics of the imagination. The IP intervention was well-accepted, easily applicable, and various effects, pill characteristics and appearances were described. While many participants did not desire a physical pill, either due to the absence of the imagination component or aversion to pills, the approach was considered to be cognitively and time demanding, which in turn, however, encouraged the establishment of a therapeutic ritual that protected against the increase in test anxiety during the preparation phase. OLP findings were comparable, and especially the importance of a treatment rationale was stressed in both groups, counteracting an initial ambivalent attitude. The RTA findings were supported by the open-ended questions of the RCT.

**Conclusion:**

IPs appear to be a well-accepted and easily applicable intervention producing a variety of beneficial effects. Thus, the IP approach might serve as an imaginary based alternative to OLPs warranting further investigations on its application to harness placebo effects without a physical pill.

## Introduction

Traditionally, placebos are tied to randomized controlled trials (RCTs) as a concealed and inert treatment (e.g., sugar pill, saline injection) to isolate the specific efficacy of a verum by controlling for therapeutic noise, such as expectancy, spontaneous remission, or regression to the mean [1]. However, placebos have moved beyond being a mere methodological tool. The placebo effect has been established as a means to gain positive therapeutic outcomes in clinical trials [1], and its utilization is widespread among doctors and medical specialists [2–4]. Nonetheless, the deceptive nature of the placebo treatment is controversial and faces multiple ethical hurdles [5]: It threatens patients’ autonomy, practitioners’ obligatory veracity, and finally patient’s trust in the therapeutic relationship [6]. In this context, the prescription of open-label placebos (OLPs; [7]) holds the promise of a treatment that harnesses placebo effects in a transparent and ethical way [8]. The clinical potential of placebos and specifically OLPs is evident and further research on their beneficial effect is being called for [9, 10].

Recent meta-analyses found medium to large effect sizes for OLP in clinical conditions [9] and a medium-sized effect in self-reported outcomes in experimental nonclinical conditions [11]. Thus, placebos also seem to work when given without deception. Yet, what contributes to the positive effects of an OLP–consisting of a pill, a rationale, and a therapeutic interaction [8]–remains unclear. Through the elimination of the physical pill, it is possible to examine whether the pill is a necessary component in producing beneficial effects.

The concept of an imaginary pill (IP) was first introduced by De Shazer in 1984 in the context of clinical hypnosis [12] and more recently, Niels Bagge, a Danish clinician, independently introduced the same idea based on the concept of placebo research [13]. To test the efficacy, we examined the effects of an IP intervention and compared it to OLP in an RCT in students with self-reported test anxiety. We found comparable positive effects for IP and OLP with a medium to large effect size (*d* = 0.71) in comparison to a control group [14].

However, apart from intervention efficacy, the promising findings on IP warrant qualitative research to include participants’ perspectives regarding the interventions’ acceptability (i.e., credibility, trust, and belief), applicability and the experience of effects of the IP. In the field of OLP research, several qualitative studies have already been conducted wherein it was found that participants have a mixed reception to the OLP treatment idea, with reactions ranging from skeptical to curious and hopeful [15–21]. For instance, irritable bowel patients receiving OLPs were more ambivalent and self-reflective as compared to those in the deceptive placebo group [15], whereas women with menopausal hot flushes had an overall positive experience with the OLP treatment [21]. In contrast, participants in an experimental pain study were rather skeptical about the efficacy of the intervention, despite beneficial treatment effects [20]. This might be explained by a lack of trust in the competence of the providing health care professionals, as well as perceived self-efficacy in solving a problem, as Druart and colleagues’ qualitative study on experimental pain showed [22]. In postoperative pain, patients perceived an OLP both as trustworthy and ethical [23], but experiences and perceptions of the treatments’ efficacy appear to vary widely [16]. A large range of reactions is also displayed by physicians reviewing the idea of OLPs [4]. Hence, an additional qualitative study in this research field may expand the current database and include participants’ experiences of a placebo intervention without a physical pill.

The aim of this embedded qualitative study was to generate initial knowledge of views and experiences toward the novel IP intervention and to compare these views with the experience of individuals who received OLPs (*N* = 20). Further, we sought to corroborate these findings with data from open-ended questions of the RCT (*N* = 114) and to provide insights into individual characteristics and appearances of imagined pills from RCT participants.

## Methods

### Study design

This qualitative study was embedded within an RCT testing the efficacy of IP and OLP interventions compared to a control group in reducing participants’ test anxiety over three weeks [14]. In short, participants were randomly assigned to one of the three groups and received their intervention in an online treatment session. During this session, both intervention groups received a treatment explanation (i.e., rationale) and participants in the IP group practiced taking their IP together for the first time with the assistance of the treatment provider. Both intervention groups were instructed to apply their respective placebo procedure twice daily for three weeks. After study completion, randomly selected participants were contacted via email and provided with relevant information regarding the planned qualitative study. Upon receiving informed consent, semi-structured interviews were scheduled and held online (due to pandemic restrictions). The study design and informed consent was approved by The Ethics Committee of the Faculty of Psychology, University of Basel, Switzerland, and was carried out in accordance to the principles expressed in the Declaration of Helsinki. The study was registered at ClinicalTrials. gov: NCT04250571 (31/01/2020). Access to study data was limited to the study personnel and all identifying data was anonymized. This qualitative study was reported in accordance with the standards for reporting qualitative research (SRQR) checklist [24].

### Study participants

Of the 114 participants in the intervention groups of the RCT, 20 participants (i.e., OLP n = 10 and IP n = 10) were randomly selected to take part in the qualitative study. In case participants either did not want to take part in the study or did not respond to the recruitment request, the next person on the randomized list was contacted until ten participants per group were reached. The sample consisted of students at the University of Basel, who had an exam at least four weeks ahead and self-reported suffering from test anxiety. The students’ test anxiety was within the normal range of anxiety scores (below 60), indicating that our participants were a non-clinical group with average test anxiety scores.

Being a master’s student, insufficient German skills, and problems swallowing pills were reasons for exclusion. Incentives to take part in this nested qualitative study were credit points or a fixed monetary compensation (20 Swiss Francs). Recruitment took place between June 2020 and June 2021. Table 1 depicts the sociodemographic characteristics of the 20 study participants.

**Table 1:** Sociodemographic characteristics of the study participants.

Group	N	Age (±SD)	N (%) female	Mean interview duration in minutes (±SD)	N (%) Psychology student
**IP**	10	24,5 (±5,5)	6 (60%)	42,2 (±9,3)	10 (100%)
**OLP**	10	22,9 (±5,2)	8 (80%)	37,1 (±7,5)	9 (90%)

*Note*. *IP* imaginary pill

*OLP* open-label placebo

*SD* standard deviation.

### Interview procedure

The semi-structured interviews were conducted by SB. The platform used was zoom (), which allowed the audio recording of the session. The interviews aimed at receiving a comprehensive insight into the subjective views and experiences about the interventions. It was stressed that there are no right or wrong answers and that participants can talk freely and criticism or suggestions for improvement of the intervention are welcome. In some cases, non-predefined questions were asked for comprehension issues and for an overall agreeable and open conversation atmosphere. The interviews included questions about the following study time points: (a) the treatment session, (b) the three-week intervention phase, (c) the exam situation, and (d) possible future use. Interview questions only varied slightly between IP and OLP participants and can be found as supporting information (S1 Appendix: interview questions). The interviews were conducted in Swiss German and transcribed verbatim in the German language with preservation of typical Swiss German expressions, following an integrative approach [25]. Quotations in the results were translated from German to English.

### Qualitative analysis

The interviews were exported to and analyzed with the software MAXQDA (https://www.maxqda.com). A reflexive thematic analysis (RTA) approach [26, 27] was employed. We deviated from the originally planned qualitative content analysis by Mayring as the RTA is more commonly used in the English-speaking world and has previously been adopted in several qualitative OLP studies, which allows for better comparability. The number of interviews (*N* = 20) was considered as sufficient according to practical guidelines [28] and allowed us to reach a desired ‘saturation’, where no new information was generated by more interviews. An inductive-deductive hybrid approach was used to analyze the qualitative data [29]: First, an inductive and data-driven coding process was used to map the content of the interviews for a phenomenon with limited research literature as accurately as possible [30–32], and in a next step, a more deductive approach was used to generate themes that were relevant and meaningful to the research question [33]. With the aim of developing a coding system and key themes across the interviews, the following steps were performed: (1) Prior to coding, two study team members (AB, ME) familiarized themselves with the dataset by reading through the interviews thoroughly. (2) Initial codes were generated by AB and ME. The codes (i.e., short segments of the interview conveying significant information about the topic) were chosen based on their perceived meaningfulness and relatedness to the research question. To maintain the characteristic features of IP and OLP interviews, two unique code systems were developed. (3) To minimize bias, 50% of the interviews were coded by two independent coders (AB, ME; [34]). Codes of the independent coders were regularly compared, and the code system was updated based on agreement. (4) A common consensus about the code system was found in regular group meetings, including SB and DS. (5) Codes were grouped by AB and ME into categories and these further into main categories. These steps were done based on inductive similarity and belonging of codes and deductive relatedness to the research question. (6) Based on the main categories, initial themes for both code systems were formed by ME. As a result of their congruence, both code systems (i.e., IP and OLP) were merged. (7) Upon further revision of the themes, the final system was created by AB and SB.

### Qualitative analyses of open-ended questions from RCT and imaginary pill characteristics

In order to back the qualitative analyses, open-ended questions completed at the last assessment time point of the RCT were further included in this analysis (S2 Appendix: open-ended questions of RCT). This sample included all OLP and IP participants of the trial (*N* = 114). Results of two open-ended questions (i.e., “Why did you find the explanation that the IP/OLP can work helpful?”; “Did you assume that the IP/OLP would work or were you skeptical?”) were included in the current analysis. Further analyses on open-ended questions, such as treatment credibility and intervention idea or learnings due to study participation, can be found in the supporting information (S1-S3 Tables: open-ended questions in S1 File). All of the responses were imported to the online software MAXQDA2022 and a descriptive method of data analysis was used [35]. Data analysis was conducted by thematically coding participant’s comments, without a thorough theme analysis [36]. Two coders (BB, CB) repeatedly read the comments to achieve familiarization and then used an inductive, open coding process in which descriptive labels (i.e., codes) were assigned to each comment. Both coders worked independently, and for comments that communicated numerous meanings, several codes were used. Next, codes were examined and compared to find commonalities and discrepancies. To offer a comprehensive overview of the comments, similar codes were sorted into higher-level categories.

Since the IPs were in participants’ minds and not visible to observers, we wanted to capture the inner experience and characteristics of the participants’ pills including their appearances with graphical illustrations. To do so, all IP participants in the RCT had to complete an interactive document to describe the characteristics of their pill (type, shape, color, packaging, size) and its effects immediately after the treatment session. The frequencies of each feature and effect were summarized in a table. Nine IPs were selected and illustrated by BB using the software blender 3.2.2 (https://www.blender.org).

## Results

### Qualitative analysis

Of a total of 37 contacted, 17 (OLP n = 8 and IP n = 9) did not respond to the recruitment request or declined to participate. The twenty study participants who took part in the interview had a mean age of 23.7 (± 5.4) years, 70% of them were female and 95% psychology students (see Table 1). The interviews had an average length of 39.4 minutes (± 9.1).

The interviews consisted of 106’946 words in total. Based on this material, 816 codes were generated, of which 651 were used for the analysis. The codes were summarized into 36 categories, which in turn were subsumed to 14 main categories. These categories were integrated into 4 key themes: (1) attitude towards the intervention, (2) applicability of the intervention, (3) experience of effects and (4) characteristics of the imagination. Due to congruent content between the IP and OLP coding systems, theme 1 to 3 capture both interventions, while theme 4 relates only to the IP group. Table 2 presents a detailed presentation of the key themes and main categories, including all listed categories. In the following, key themes and corresponding main categories are presented with a selection of relevant quotations.

**Table 2:** Key themes with underlying categories.

Key themes	Main categories	Categories
**Attitude towards the intervention**	Acceptance	Approval of the intervention
		Open attitude towards the interventions
	Expectation	Expectations towards the interventions / How expectations were met?
	Skepticism	Doubts towards the interventions
		Attitude towards IP from the OLP group
		Importance of the rationale for trust in OLP
		Importance of the treatment session (e.g., pill intake) for trust in IP
**Applicability of the intervention**	Preferences of pill intake	Schedule of the pill intake
		Ritualization of the pill intake
		Desire to individualize the pill intake
		Desire for physical placebo
	Integration of the pill into daily life	Difficulty to remembering pill intake in daily life
		Easy integration of pill intake into daily life
		Difficult integration of pill intake into daily life
		Reminder e-mails help with integration into daily life
		Intervention requires cognitive effort
	Application over time	Pill intake was more difficult over time
		Pill intake was easier over time
		Motivation-changes over time
		Refresher-appointment would be helpful
**Experience of effects**	Increased self-confidence	Increased self-awareness/-efficacy
		Increased self-confidence
	Stress relief	Stress reduction and increased relaxation
	Increased concentration	Increased mental focus/concentration
	Reaction to IP effect	Astonished that IP works *[only coded in IP group]*
	Modulating factors on the effect	Positive influence of a routine
		Effect dependent on the daily context
		Effect dependent on exam proximity
		Effect-changes over time
	Effects on the exam	Positive effect on learning
		Positive effect on examination situation
		Uncertainty about the effect on examination situation
		No effect on examination situation
**Characteristics of the imagination**	Individual aspects of imagination	Individual aspects that supported the imagination
	Realness of imagination	Body sensations during imagination
		Vivid imagination

### Theme 1 Attitude towards the intervention

#### Acceptance

The IP intervention found wide acceptance among participants and various reasons were expressed why this treatment felt right for them. The imagined pill intake was described as easy and effortless, taking only a few minutes. It was viewed as a new and interesting idea, practical and flexible in its use. Also, participants expressed that the IP intervention was helpful and efficacious, and one participant preferred the IP over OLP due to reluctance to take real pills. The level of approval with the assigned intervention was also high among OLP participants. Example responses include:

” […] actually, I was amazed that you can have such an impact in this short amount of time.” (IP; Subject 14)

“No, so for me [taking a physical pill] would have been bad. […] So, I liked it much more [than OLP] since it was imaginary.” (IP; Subject 17)

“I kind of always looked forward to it, it was always like: ‘Uh, you still have to take your placebos.’” (OLP; Subject 1)

The pills (imaginary and physical) were associated multiple times with a drug or a symbol for healing or health improvement. It was emphasized that people are socialized with pills in their culture as a mean to get better and one participant reflected on the common familiarity with them:

“[…] yes, the [imaginary] pill strongly resembles a drug, and I would say that most of the people have already taken a drug in their lives. So, it’s a good choice, a good method.” (IP; Subject 15)

Many approached the IP intervention with an open attitude. Participants showed a mixture between curiosity and excitement about the previously unknown approach. Some employed a mindset of ‘let’s give it a try’, as there were no costs or harmful side effects involved.

Contrary to IP, half of OLP interviewees had pre-existing familiarity with (open-label) placebos. Regardless of prior unfamiliarity, however, there was an openness and curiosity about the intervention:

“I have heard about placebo before–the placebo effect–and that studies are done about it. But I didn’t know that you can also do that with open placebos, that you can disclose that. I had never heard of that before. I actually found that exciting, that it is being tried out like that.” (OLP; Subject 9)

Approval towards the IP intervention was also apparent as the majority of participants showed openness to reapplying the intervention for the next exam phase in the same or a slightly altered way. Moreover, nearly all interviewees reported that they could imagine an extension of the IP intervention beyond test anxiety. Especially regulation of other emotions (insecurity, sorrow) as well as pain were regularly mentioned to be possible areas of application. Most participants would furthermore recommend the intervention to others, however, only to people that show open-mindedness:

“To certain people in my environment, I would definitely recommend it, yes, others, definitively not, because, well, I think that it is something that you really have to make up your mind to do it and believe in it.” (IP; Subject 16)

These results are consistent with those of the OLP group, in which open-mindedness was also considered a prerequisite for the application of an OLP intervention.

#### Expectation

Most participants’ expectations of IP were met. Seven participants reported that their expectations were fulfilled, with three of them claiming it was exceeded. Some, however, reported initial skepticism and therefore lower expectations, which were then easier to be met:

“I would say, exceeded, yes. Well, simply because of the skepticism that I had in the beginning. Or I underestimated that it can really work, maybe it is better to say it like that.” (IP; Subject 16)

Similarly, almost all OLP participants reported fulfilled expectations. Again, initially low expectations were existent in this group:

“I must say almost more than I would have thought. […] Somehow, I had the feeling my anxiety is too big that it could have a big effect. But in the end, I think it helped me and gave me a certain security, also in myself. And from that point of view, I would say it was more than I expected initially.” (OLP; Subject 8)

#### Skepticism

Many participants in the IP group reported that they felt skeptical or doubtful about the intervention, especially in the beginning. While some participants expressed insecurities towards an unknown method, others mentioned doubts regarding the required resources (e.g., time, cognitive capacities). Comparably many OLP participants expressed initial skepticism.

When asked about the other group’s intervention, a majority of IP participants preferred taking a pill the imaginative way. A physical component was not wished for, either due to the absence of the imagination component (which they liked), or because of a reluctance to taking real pills. Further, two IP participants expressed that the imagination was satisfactory and helpful, thus not wishing anything to be different:

"So, it did help me, the imaginary pill, so I don’t know if a real placebo would have helped more than this." (IP; Subject 18)

[about whether OLP would have been better]”I don’t think so, because then the imagination would have disappeared. I don’t know what would have happened if I had taken a Tic Tac […] but ehm, I think the pure imagination that I needed for it, would then have vanished. And that’s why I found it really cool that it is all about imagination, yes.” (IP; Subject 16)

However, feedback from six IP participants indicated that the imaginative method is cognitively taxing and requires concentration and calmness. Three of those interviewees thus expressed that, even though the IP worked for them, taking a physical pill might have been easier and less tiring. On the other hand, eight participants in the OLP group expressed openness and interest toward an IP intervention, yet, assumed that an imagination might be more challenging. In this context, three participants also stated that they preferred taking a physical pill.

[about taking an IP] “Yes, I mean the critical part in me says: […] ‘I need a physical correlation here, something that someone sends me, what someone has packed, what is produced […] It would probably be more difficult for me to believe in it.’ […] And then there’s another part in me saying: ‘Mind over matter.’ […] Just because you can touch it […] doesn’t really make any difference." (OLP; Subject 1)

The treatment session where participants received the intervention reduced skepticism and strengthened the trust in the intervention. Almost all IP participants reported that–besides the explanation–the joint IP intake was helpful, fostered trust, and countered their initial skepticism. The comments of the IP participants showed that the single practice with the provider within the RCT was enough to gain security to continue the practice independently.

“This exercise that was done together with the pill taking was kind of really cool, because I had this feeling of ‘huh that really works?’ […] I wouldn’t have expected that before.” (IP; Subject 12)

Along these lines, almost all OLP interviewees were convinced by the rationale, which helped to reduce skepticism at the beginning and during the intervention. The most memorable and convincing discussion points in the treatment rationale were that the effects can occur even with a skeptical attitude (n = 5) and that a possible mechanism is assumed to be conditioning (n = 2), which can be explained by an automatic biological mechanism (n = 1).

“So, the biological [discussion point] made sense for me, with the neurotransmitters, I think that’s the one that kind of sold me the most and that’s also the one that I thought about the most during the study as well.” (OLP; Subject 5)

“I believe that an additional thing was that it was said that placebos also work, even if you don’t believe in it. Simply taking the pills can help. And I think that was something that was most notably decisive.” (OLP; Subject 6)

### Theme 2 Applicability of the intervention

#### Preferences of pill intake

An external structure was provided to the participants for the pill intake, that comprised daily reminders in the morning and evening, encouraging a fixed intake schedule. The majority of IP participants preferred this method over a variable pill intake schedule (i.e., on demand intake). Mainly because a fixed schedule supported ritualization, reduced forgetfulness, and prevented negative feelings (stress, nervousness). Only a few participants voiced a preference for a pill on demand. The voiced preferences were similar in the OLP group.

“I also don’t think that you can build up a routine just like that for a few days. Instead, I think it takes a little longer […]. I am rather of the opinion that you don’t use it in an acute way, but rather for a longer period of time. Maybe that it just becomes like a part of life itself.” (IP; Subject 15)

#### Integration of the pill into daily life

Around half of the participants in both groups combined the pill intake with a form of ritualization. This was either an integration into an existing ritual (morning routine, breakfast, etc.), a link with a suitable situation (start of the learning session, break during learning), or simply with drinking a glass of water. The latter was even present in the IP group, where three participants either drank a glass of water or performed swallowing motions when taking their IP, supporting the imagination:

“I actually had to make a swallowing motion to make it really go down in my head. Well, you were allowed to do that, and I didn’t take anything else. I had to do something so that I felt that it’s really something real.” (IP; Subject 20)

Three out of four IP participants who did not integrate the pill intake into a ritual reported that it might have been helpful to do so. One of them explained that the intervention would have been easier if repeatedly done in the same context, one that is preferably quiet. Another IP participant, who embedded the IP intake within a ritual, reinforced the need for such a ritual:

"It’s supposed to be a kind of ritual. If you just swallow a pill, it works for maybe three seconds, or not even, I don’t know. And from my point of view, imagination is something that, if you don’t do it often and practice it, is just […] it needs a calm mood. You can’t just say: ‘Now I’m at the train station and pop this imaginary pill’, it has to be an environment. It takes a lot more time, I think, for you to really feel this effect. […] It needs such a ritual." (IP; Subject 19)

Various reasons were conveyed about challenges to integrate the IP into daily life. Also, a few participants sometimes forgot to take their IP. These difficulties were also mentioned in the OLP group. However, the need for cognitive effort while taking the pill was solely emphasized in the IP group.

#### Application over time

The time course affected not only the experienced effect of the IP but also its application. For instance, a participant shared that motivation for the pill intake dropped after some time due to a lower level of suffering. However, motivation for applying the intervention also increased when it was experienced as helpful. In addition, five participants in the IP group expressed a wish for a so-called ‘refresher-appointment’ (i.e., a second meeting in the middle of the intervention phase that would renew the placebo information and the IP exercise). They explained that this would be helpful for remembering the rationale, to consolidate and remember the desired feeling connected to the IP, and to generally increase motivation. Besides the interactive document in which participants recorded their IP, a few participants suggested ideas for alternative or idiosyncratic reminders to recall specific placebo information. For instance, one IP interviewee proposed having an auditory aid:

“But maybe also an idea would be, if you had that [the suggestions about the IP intake] as an audio recording or something, so instead of a refresher, on the phone or in person, you could just listen to that again.” (IP; Subject 14)

While the timing also affected the experienced OLP effect and its application, only two participants in the OLP group mentioned the idea of such an additional appointment.

### Theme 3 Experience of effects

#### Increased self-confidence and concentration, stress relief and reaction to IP effect

The IP participants reported increased self-confidence and concentration as well as decreased stress as beneficial effects during the 3-week intervention phase. Participants who reported an increase in self-confidence experienced more optimism or could draw strength and courage and thus felt supported through the pill. Others reported a feeling of security and saw the intervention similarly to an anchor that prevented the emergence of more negative feelings:

"So, it felt less that it had an effect, but more that it actually prevented other things from being triggered. […], that it just didn’t develop as strongly. So, because the stress with me is rather high, the closer the exam comes. And the feeling didn’t really come at all." (IP; Subject 13)

Stress relief was among the most prominent positive IP effects leading to increased relaxation and calmness, as well as a reduction of anxiety and stress. This was felt on a psychological (e.g., less nervous thoughts) and a physical level (e.g., general relaxation and loosening, reduction of shaking). At the same time, an increase in concentration during the learning phase was reported. Here, clearer thoughts, wakefulness, better focus, and increased inner calmness were described by roughly half the participants. The same kind of effects were mentioned in the OLP group.

Besides the mention of the beneficial effects, eight participants of the IP group were positively surprised, astonished, or impressed by those effects. These reactions were usually connected to the initial skepticism and novelty of the intervention and the unfamiliar realization that the mind can have such an effect on the body:

“So that placebos work, I already knew that before. But I never tried it myself, because I never took placebos myself and then I was positively surprised that it can work so strongly, that what the mind–stupidly said–can simply do.” (IP; Subject 16)

#### Modulating factors on the effect

The strength of the IP’s effect varied based on different factors: Most importantly, daily fluctuations in sleep, stress levels, and fatigue, or even simply the time of day sometimes made pill intakes more difficult and, as a consequence, the pill effect less strong.

“This [strength of the effect] actually depended on the day. I think when […] I had a stressful day or so, then it was difficult for me to sit down and concentrate […]. Yes, so I just think that it depends very much on the day.” (IP; Subject 12)

“It depended a little bit on how I had the space, how I took my time and how much I could focus. And if, for example, I left the house and was doing something and then: ‘Oh well, I still have to take the pill!’ then I took it and then it [the effect] came much less strongly, but if you are almost meditative, or you really take time to experience the ritual completely, then it has a much stronger effect.” (IP; Subject 19)

OLP participants reported that the exam proximity changed the experience of effects (see also *application over time* in theme 2). With the increase in pressure and nervousness due to the approaching exam, four respondents showed enhanced motivation for the pill intake and increased effects of the OLP:

"As the exam was approaching, you became more nervous and you needed something that would help you [..] so it was really like that, the need increased and the effect also went up." (OLP; Subject 2).

On the other hand (and similar to IP participants who reported the need for a refresher appointment as the effect diminished after the treatment session) some OLP participants reported a decrease in effect due to an increase in skepticism, which in turn negatively impacted motivation:

"Well, at first I believed in it, in the beginning, let’s say the first week, I was kind of motivated to do it,… it’s just that afterward, I believed in it less, I became more and more skeptical, I thought about it more, yes." (OLP; Subject 5)

#### Effects on the exam

Participants had mixed perspectives on the pills’ effect during the exam situation. In the IP group, nine participants were either unsure if the pill had a positive effect on the exam itself or thought it might have been subliminal. Some felt less nervous and more calm compared to previous exam situations, but could not certainly attribute it to the pill. Half of the IP participants expressed that they rather felt a helpful and buffering effect during the learning phase but not during the exam.

Similarly, five OLP interviewees thought that the intervention helped indirectly for the exam by facilitating the learning phase. For the other five participants, the OLP induced noticeable effects during or right before the examination that ranged from better mood, increased calmness to reduced anxiety.

### Theme 4 Characteristics of the imagination

#### Individual aspects of imagination, realness of imagination

All IP respondents described a vivid and detailed imagination, which was maintained during the intervention phase. Concerning the pill, the majority of participants imagined characteristics of the pill that resembled real pills, including form, size, color, and taste. Participants were also able to regularly reproduce the desired state connected to the pill:

“I found it impressive how I was able to revive this feeling from this situation that I imagined [during the treatment session]. That I truly felt it for real and that it continued to work later when I took the imaginary pills [by myself].” (IP; Subject 14)

"It was like a mental picture, but I could really picture it quite well, so like the color, shape, size and then also the taste in the mouth and also the physical effect, so really quite quite well." (IP; Subject 19)

Five respondents reported having found it helpful to link the desired effect of the IP with the positive situation identified during the treatment session. For example, some participants focused on specific situations, such as playing an instrument (e.g., ‘when I take the IP, I want to feel the way I feel when I play the cello’) or more complex imaginary scenarios, by being mindful and focused at present:

"So, I always imagined it like this, as if I would now go from the desk in my room to the kitchen and open the drawer, take out the pill, have it in my hand, put it in my mouth and then take a sip of water. I really imagined myself doing the movement but just stayed at my desk. […] Each time I could say to myself ‘I’m going to take a little round pill that’s bright yellow. It has no smell, no taste’. I think that helped me to make this mental journey, to imagine the whole way to the kitchen every time, every step." (IP; Subject 18)

Remarkably, some participants had physical sensations in their bodies during their IP intake. Based on suggestions during the exercise in the treatment session, one participant felt the imagined pill sliding down her throat. This sensation reinforced the trust in the pill for the interviewee, who was surprised that an imagination can induce such a bodily feeling. Other participants reported experiencing side effects such as a dry mouth, goosebumps or warmth radiating from the abdomen. These side effects were suggested during the pill intake in the treatment session to demonstrate the IP response and were in fact found to be helpful in convincing people of the pill’s efficacy. Overall, realistic and detailed imagination were regularly reproduced and maintained during the three weeks of intervention:

“[…] when I thought of it, it didn’t take a long time for the image to appear, but it appeared like when I would think of a piece of paper or of a pen as if I had already had it for real or seen it before [… .]. So, if I would draw it now, I’m not good at drawing, but if I had to draw it, I would be able to.” (IP; Subject 11)

### Qualitative analyses of open-ended questions from RCT and imaginary pill characteristics

To corroborate the RTA, two open-ended questions from the RCT were analyzed qualitatively. A total of 221 responses to the two questions were received from 114 participants, with the responses typically being brief comments or phrases. Participants’ characteristics can be found in the main study [14]. In both groups, the most commonly cited reasons why the treatment explanations were helpful were that ‘the explanations made sense’, ‘led to a better understanding’, ‘created belief’, and ‘strengthened previous knowledge/belief’ (see Table 3). Table 4 depicts the responses to the question concerning skepticism towards the efficacy of the received intervention: While ‘belief in the effect’ was frequently expressed, a comparable number of statements included skepticism.

**Table 3:** Helpfulness of explanation: Why did you find the explanation that the imaginary pill / open-label placebo helpful?

	OLP	IP	Total
	N = 78	N = 76	N = 154*
The explanation…	N (%)		
**… created faith**	**10 (12.8)**	**8 (10.5)**	**18 (11.7)**
… gave new knowledge	7 (9)	3 (3.9)	10 (6.5)
**… led to better understanding**	**13 (16.7)**	**9 (11.8)**	**22 (14.3)**
… was helpful	5 (6.4)	1 (1.3)	6 (3.9)
… was believable	3 (3.8)	2 (2.6)	5 (3.2)
… made imagination easier	5 (6.4)	4 (5.3)	9 (5.8)
**… strengthened previous knowledge/beliefs**	**7 (9)**	**9 (11.8)**	**16 (10.4)**
… mentioned previous studies	5 (6.4)	7 (9.2)	12 (7.8)
… was conforming to personal interest	1 (1.3)	0 (0.0)	1 (0.6)
**… made sense**	**8 (10.3)**	**15 (19.7)**	**23 (14.9)**
… created an expectation	4 (5.1)	2 (2.6)	6 (3.9)
… focused on positive aspects	2 (2.6)	1 (1.3)	3 (1.9)
… gave security	1 (1.3)	0 (0.0)	1 (0.6)
… was credible	2 (2.6)	0 (0.0)	2 (1.3)
… left an open outcome	1 (1.3)	1 (1.3)	2 (1.3)
… showed researcher allegiance	0 (0.0)	1 (1.3)	1 (0.6)
… improved mindfulness	3 (3.8)	6 (7.9)	9 (5.8)
N/A	1 (1.3)	8 (9.2)	8 (5.2)

*Note*. Statements in bold are those that were mentioned by more than 10% of the respondents in total

*N (%) indicates the number (percent) of participants who mentioned the respective topic in their answer

Since several answers were coded per question, one person can be included several times in the data

*IP* imaginary pill

*OLP* open-label placebo.

**Table 4:** Skepticism towards treatment: Did you assume that the imaginary pill / open-label placebo would work or were you skeptical?

	OLP	IP	Total
	N = 70	N = 71	N = 142*
	N (%)		
not skeptical	0 (0)	3 (4.2)	3 (2.1)
**believed in effect**	**20 (28.6)**	**28 (39.4)**	**48 (34)**
open minded	8 (11.4)	7 (9.9)	15 (10.6)
Hopeful	4 (5.7)	2 (2.8)	6 (4.3)
Curious	1 (1.4)	0 (0)	1 (0.7)
Neutral	2 (2.9)	0 (0)	2 (1.4)
Unsure	1 (1.4)	0 (0)	1 (0.7)
slightly skeptical	1 (1.4)	7 (9.9)	8 (5.7)
skeptical at first	2 (2.9)	2 (2.8)	4 (2.8)
grew more skeptical over time	1 (1.4)	1 (1.4)	2 (1.4)
**Skeptical**	**29 (41.4)**	**21 (29.6)**	**50 (35.5)**
very skeptical	1 (1.4)	0 (0)	1 (0.7)

*Note*. Statements in bold are those that were mentioned by more than 30% of the respondents in total

*N (%) indicates the number (percent) of participants who mentioned the respective topic in their answer. Since several answers were coded per question, one person can be included several times in the data

*IP* imaginary pill

*OLP* open-label placebo.

**Fig 1:**
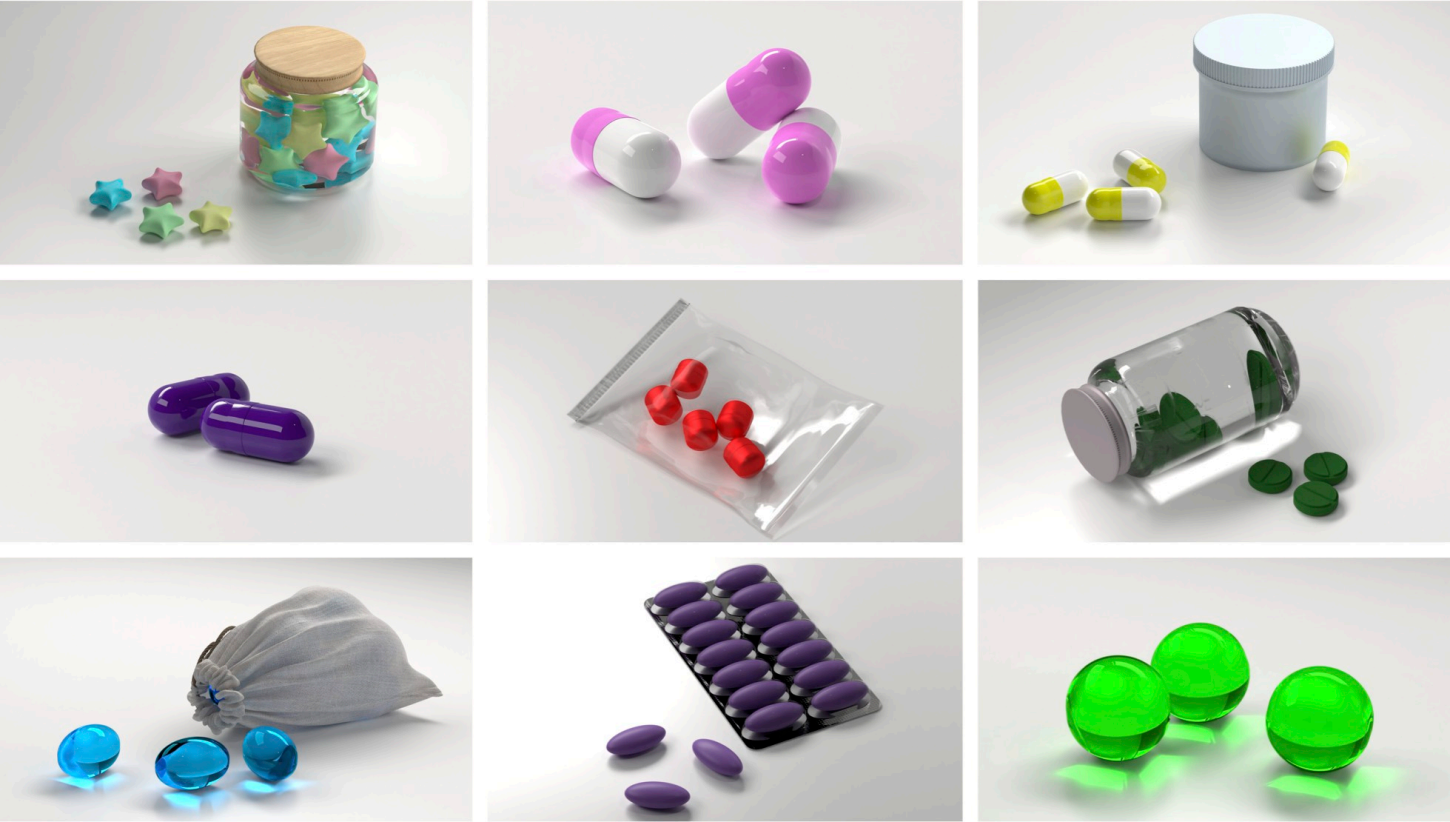
Visualization of a selection of imaginary pills. Imaginary pills of nine study participants and their packaging (if existing).

Table 5 shows the characteristics and effects of the IPs recorded in the interactive document after the treatment session: A wide range of different pill shapes, colors and packaging and particularly various effects were imagined. The most common type was a small, white pill with a round shape packed in a blister. However, more distinctive shapes (i.e., star-shaped), colors (i.e., colorful), and packaging (i.e., tins) were also reported. The most prevalent effect of the IP was ‘relaxation’ followed by ‘focus’, ‘confidence’ and ‘better mood’. Visualizations of the pills can be seen in Fig 1.

**Table 5:** Overview of the characteristics of the pills in the imaginary pill group and their frequencies.

	N		N
Kind		Effects	
Pill	39	Relaxation	49
Capsule	10	Focus	34
Lozenge	5	Confidence	14
**Shape**	** **	Better mood	11
Round	32	Alertness	5
Oval	19	Motivation	5
Globule	1	Optimism	4
Star shaped	1	No tremor	4
Octagon	1	Control	4
**Color**	** **	Stops rumination	4
White	20	Lowering pulse	3
Blue	10	Security	2
Colorful	10	Soothes intestinal problems	2
Green	4	Good sleep	2
Pink	3	Feeling of freedom	2
Purple	2	Positive stress	2
Red	2	Energy	2
Yellow	2	Competence	1
Grey	1	Light chest	1
**Packaging**	** **	Connected to nature	1
Blister	24	Narcotizing	1
Glass	11	Heightened blood flow	1
Tin	10	Less stress	1
Box	7	No sweating	1
No package	1		
Sachet	1		
**Size**	** **		
Small	29		
Medium	18		
Very small	4		
Big	3		

*Note*. Features might be mentioned several times.

## Discussion

This qualitative study–nested within a 3-week RCT in test anxious students–sought out to assess participants’ views and experiences towards a novel IP intervention and to compare them to those of participants receiving OLPs. Further, we aimed to corroborate these findings with data from open-ended questions of the RCT and to give insights into individual characteristics and appearances of imaginations from study participants.

Four key themes were identified: (1) attitudes towards the intervention, (2) applicability of the intervention, (3) experience of effects, (4) characteristics of the imagination (see Table 2). Regarding the attitude (theme 1) and applicability (theme 2) of the IP intervention, most interviewees showed a high level of acceptance and mentioned its easy application with minimal effort required to achieve a positive effect. While a few, however, expressed a preference for a physical pill because imagination requires more time and cognitive effort, the majority of participants did not desire a physical component but were intrigued by the ‘power’ of imagination. For a few participants, taking a physical pill might have caused the imagination to disappear. Participants approached the IP intervention with an open attitude and with a mindset of ‘let’s give it a try’ and ‘it won’t harm me if it doesn’t work’, the latter underlining the advantage of such placebo interventions having no known side effects. Most interviewees showed openness to reapplying the IP intervention, to extending its use to other symptoms (i.e., pain, sorrow, migraine), and recommending it to relatives and friends with open mindsets–a finding that shows that the IP offers flexibility and empowerment of users. Initial skepticism was commonly present in the IP group but seemed to decrease for some participants over the course of time. The results from the open-ended questions were consistent with this ambivalent finding: Many respondents in both groups reported being skeptical, while just as many also believed in the effect, with many expressing both (see Table 4). The treatment session seemed to be crucial to counteract skepticism: Besides the joint intake of the IP, the treatment rationale was perceived as convincing. This finding is supported by the open-ended questions, which revealed that the explanations led to better understanding, strengthened previous knowledge, made sense, and created faith in the intervention (see Table 3). In terms of IP application, the intake was often ritualized and/or embedded in a pre-existing ritual, and a preference for a fixed intake schedule was generally voiced. This preference supports the choice of a fixed schedule over a variable one and reflects the use of many (pain) medications which can be taken on a regular basis and/or on demand. Thus, the understanding of IP also seems to align with normal pill prescribing. The symbol of an (imaginary) pill itself elicited familiarity among participants, as it was described that pill-taking is embedded in their culture. Further, the IP intervention was preferred by a participant with an aversion to pills.

Regarding the experience of effects (theme 3) and characteristics (theme 4) of the IPs, a wide range of beneficial effects, as well as various individual pill properties were reported. Mostly, the IP intervention helped with stress relief and increased participants’ concentration and self-confidence. The experienced effects matched those, that participants recorded in the interactive document after the treatment session (see Table 5). The IP approach further seemed to serve a preventive function during the learning phase, partly by providing a soothing and sustaining daily ritual, some of which allegedly prevented the onset of severe symptoms altogether. The quality and intensity of the IP effects varied, as factors such as daily context, exam proximity or treatment session proximity influenced the pill intake. However, imagining the pill was perceived as easy and participants could continuously maintain the visual image of the pill which they had imagined at the treatment session. With respect to the appearance of IPs, a wide range of different pills were described (e.g., shapes, colors, and packaging; see Table 5), with the most common pill being small, round, and white, packaged in a blister. This type of pill and packaging (i.e., blister) represents a very common form in Switzerland. While more distinctive shapes, colors, and packaging were also described (see Fig 1 for a selection), it is noteworthy that the majority of IPs correspond to a conventional appearance of pills. This realistic appearance could, in turn, underpin the credibility and familiarity of the intervention.

Overall, the qualitative findings derived from the RTA could be confirmed with the open-ended questions from the RCT and the results from the IP group can be applied to those participants receiving OLPs: The OLP intervention was equally well received, described as easy to apply, and initially met with ambivalence (openness and skepticism), although initial skepticism was countered by the treatment rationale provided. Similarly, the reported effects of both groups were comparable. However, some intervention-specific themes emerged in the qualitative interviews, particularly related to only one intervention: Imagining a pill was, for instance, considered more demanding and challenging by some participants compared to taking a physical pill, whereas imagination was also regarded as a central component of the intervention. Additionally, the IP intervention was described as a possible alternative for individuals who have an aversion to pills. In terms of effects, whereas both interventions were perceived as beneficial during the learning phase, solely OLP participants additionally reported having felt a supportive effect during the exam. This finding suggests that OLPs may be more helpful in acute situations, whereas IPs may be more protective, counteracting the onset of symptoms in a preventive manner.

This is the first qualitative study on the use of IPs. However, qualitative research exists in the field of OLP: There, participants of OLP intervention arms rated OLPs to be acceptable and reported an overall positive experience [21] expressing curiosity towards this intervention [15]. Yet, participants also expressed ambivalent feelings: On the one hand, they were open about taking an OLP, on the other hand, skepticism coexisted with open attitudes [15, 16, 23]. Taken together, these results are congruent with experiences reported in the OLP group of the current study which further supports the finding that ambivalent attitudes and the cooccurrence of belief and skepticism are a common theme in OLP administration. It is noteworthy that the treatment rationales in this study addressed and appeared to mitigate skepticism successfully. Especially the explanation of underlying mechanisms (e.g., conditioning) as well as the information that an open attitude helps but is not necessary were most remembered and perceived as convincing. In line, a recent survey identified the mention of classical conditioning and brain mechanisms within the OLP rationale to be perceived as plausible [37]. However, these findings are contrasting the ones of Locher and colleagues [20], where participants seldom emphasized classical conditioning as a mechanism of placebo effects. Instead, other factors such as general attitudes and beliefs were voiced. A potential reason for this discrepancy might be that our sample was academic– 100% students and 95% of them bachelor psychology students–and thus more familiar with learning paradigms, such as conditioning. As a consequence, the biological explanation might have been most meaningful and credible due to their mindset rooted in natural science. Similarly, the survey sample by Smits and colleagues was highly educated and of relatively young age. Nevertheless, mindsets such as ‘I have nothing to lose’, ‘let’s give it a try’ or ‘it won’t harm me if it doesn’t work’ (a Swiss German saying) were in our study equally used to approach an OLP as was the case within the study of Locher and colleagues [20]. These mindsets are related to open-mindedness and the idea of hope. Hope is a factor that has already been suggested to be important in OLP effects [19], as patients, who had no previous success with medication for their symptoms, could adopt a try-out attitude of ‘what if it helps?’ [15]. This heuristic of ‘losing little if it doesn’t work, gaining a lot if it does’, which seems to be used frequently, could be an additional rationale-perspective next to ‘an open attitude helps, but is not necessary’. Overall, the current findings highlight the importance of the treatment rationale, which is not only important for the building of expectations (thus, leading to higher effects)–studied in several quantitative OLP trials [15, 38–41]–but is also important to counteract skepticism. Our findings further underline the need to adapt the proposed explanation for future studies, possibly with new and updated OLP mechanisms that resonate with participants’ beliefs [20].

Whereas the treatment rationale seems to be decisive in order to produce placebo effects, the component of a physical pill might apparently not be. IP and OLP are, however, not only equally efficacious (difference n.s. *d* = 0.11; [14]) but, as this qualitative analysis shows, both interventions are similarly well-accepted and easy to apply. Yet, IP offers an advantage over OLP in that it does not require a physical pill to be taken, reducing cost, facilitating accessibility, and increasing customization, thus providing flexibility and empowerment to users. This well-accepted approach could therefore serve, for example, as a viable solution for healthcare providers faced with unclear regulatory requirements or lack of guidelines regarding the administration of OLPs, an issue raised by US physicians [4]. Besides that, IP can be an appropriate intervention for individuals who have difficulties taking pills, a particularly underestimated issue that affects many individuals [42], some of whom not only resort to non-adherence but also alter their medication regimen, potentially compromising safety and efficacy [43]. Nevertheless, the intake of IPs can be demanding, requiring time, rest, and resources. Consequently, this approach may be more appropriate for a clientele that has mental and physical capacities, and that is not too impaired by symptoms. The potentially challenging and time-consuming part of imagination, however, was also viewed as a central part of the IP approach and thus crucial for producing beneficial effects. Therefore, the higher demands in IP have both a negative and a positive aspect: On the one hand, IP has its costs (i.e., time, and resources), on the other hand, the imaginative ritual and the small break it creates in everyday life can have a positive effect in and of itself. Thus, both the moralizing treatment explanation and the therapeutic ritual may be central to the efficacy of IP [44]. Whether these results would be reproducible at all and in a sample with fewer available resources (e.g., in an inpatient setting)–not only in terms of efficacy but also acceptability and applicability–should be investigated in future research.

### Limitations

The current study is subject to certain limitations. First, a sample consisting of young, female, and academic participants limits the generalizability of the findings. Higher education is related to more placebo knowledge [37] and may lead to expectations influencing the placebo effect. Likewise, it is possible that only those participants who also had a positive experience with the intervention responded to the interview request, ruling out potential negative views (i.e., 17 out of 37 contacted did not respond/declined). In addition, psychology students may be more responsive to such procedures than other student groups. Second, the nature of the reflexive thematic analysis involves an inductive approach and thus the researchers’ subjective interpretation of the data, which might lead to biases and inconsistencies. However, the double coding and the continuous exchange in the research group partly addressed those concerns. Third, the interviewer (SB), who was the principal investigator of the RCT and is conducting research on placebo effect, might have influenced participants’ way of answering questions. However, it was stressed that skepticism was welcome and that there are no right or wrong answers and an overall agreeable and open conversation atmosphere was created. Nevertheless, a certain social desirability effect on the sides of the interviewees might have changed some answers in favor of the treatments. Fourth, the qualitative results in this study were not compared with the quantitative findings of the RCT, i.e., it is unclear whether the subjective statements are consistent with data obtained from the RCT. Lastly, a sample size of twenty is considered small in quantitative research. However, in the case of a reflexive thematic analysis, it can be considered sufficient, and it most likely reached the state of ‘saturation’ [45], enabling a detailed and meaningful analysis of participants’ perspectives on IP and OLP treatments.

### Conclusion

The present qualitative study is the first to provide insights into participants’ views and experiences on a novel IP intervention against test anxiety. Overall, the IP intervention was well-accepted (theme 1), easily applicable (theme 2) and manifold effects experienced on a physical, mental, and emotional level (theme 3) as well as characteristics of the imagination (theme 4) were described. Whereas some IP participants did not wish for a physical pill, either due to the absence of the central component of imagination within this intervention or because of a reluctance to real pills, others viewed the IP intake as cognitively and timely demanding, requiring concentration and calmness. This, on the other hand, promoted the therapeutic ritual, which was perceived as supportive and protective against the increase in test anxiety during the preparation phase. The OLP findings were in principle comparable to those of the IP group regarding overall acceptance, application, and effects. While IP effects, however, were mainly perceived during the learning phase, OLP effects increasingly appeared during the exam itself. In both groups, initial openness to the intervention was often accompanied by a certain amount of skepticism, while the treatment rationale was seen as important to counteract this ambivalence and build trust in these (new) interventions. Hence, the IP intervention seems to be an accepted, easy-to-apply, and cost-effective intervention and might serve as an imaginary-based alternative to OLPs. This warrants further investigations on IP application in both anxiety-related domains, as well as for other clinical and nonclinical conditions to harness placebo effects without a physical pill.

## Supporting information

S1 Appendix Interview questions. (PDF)

S2 Appendix Open-ended questions of RCT. (PDF)

S1 File Open-ended questions. (PDF)

S2 File (PDF)

S3 File Data from open-ended questions of RCT. (PDF)
