# Supplementary material for: A qualitative study of imaginary pills and open-label placebos in test anxiety
Source: PLoS One. 2023 Sep 1;18(9):e0291004. doi: 10.1371/journal.pone.0291004 (PMC10473493; doi:10.1371/journal.pone.0291004)
Supplement: S1 Appendix — (PDF) [file pone.0291004.s001.pdf]

S1 Appendix: Interview questions

OLP group

---

1. <u>Intervention</u> [Treatment 3 weeks prior to the exam]

Teaser

Okay, then I'd like to start by asking you a few questions about the intervention three weeks before the exam with *** [person who made the video call]:

- What do you remember most about it?

Rationale

We gave you an explanation why placebos may also have an effect if administered openly.

- Can you remember the explanation? Can you tell me what you remember? [Complete if necessary: Discussion points to OLP etc.]
- Does this explanation make sense to you?
- What about the explanation made sense to you, what didn't?

2. <u>Intervention phase</u> [Treatment during 3 weeks]

The next questions refer to the three-week period before the exam during which you took the placebos.

Pill intake

- Did you find it easy to take the placebos? Tell me about it.
- Did the daily reminder emails help you to remember to take it as prescribed or would you have thought of taking it the anyway?
- Have you linked the intake with a ritual (e.g. breakfast in the morning)? If so with what was it?
- Was there a general feeling or a recurring thought when taking the pills? If so, what was it?
- Would you have wished that the placebo pills were not to be taken at fixed times, but rather in situations where you would have needed them more (before learning or in case of increased anxiety)?

Effect of the pill

- Did you believe in the effects of the pill or were you skeptical about them?
- When you took the placebo, did you sometimes remember the explanation given during the intervention appointment? If so, what about it? If not, why not?
- During intake, did you think about the effect?
- Do you feel that the pills have helped you during the examination phase?
    - With regards to physical symptoms, thoughts or emotions?

Pill as such

- Well, you got blue mid-sized placebo pills for your test anxiety. Is this kind of pill credible to you, or/and do you think there is another kind of pill that would have helped you better?

3. <u>Exam Situation</u>

Effect of pill

- How did you feel during the exam?

- Do you think the pill helped in the exam situation?
    - With regards to physical symptoms, thoughts or emotions?

4. **Future use**

Assessment of effectiveness and future behaviour

With regards to a future use of placebo:

- Would you use this method in a future situation where you need help (be it again before an exam or any other difficult situation)? If yes: Why? If not: Why not?
- Would you recommend this method to others? If so, to whom and why to this person?

Changes in the understanding of placebo through the study

You gave us a definition of placebos at our treatment appointment a few weeks ago. I looked it up and I'm going to read it to you right now:

"Definition of placebo given during treatment appointment"

- If you hear this definition now, would you define placebos as such again, or would you change something about it? If yes: What?
- Would you say that this study has changed your understanding of placebo? If yes: What?

IP group [red indicates differences to OLP]

---

1. **Intervention** [Treatment 3 weeks prior to the exam]

Teaser

Okay, then I'd like to start by asking you a few questions about the intervention three weeks before the exam with *** [person who made the video call]:

- What do you remember most about it?

Rationale

We gave you an explanation why imaginary pill may have an effect.

- Can you remember the explanation? Can you tell me what you remember?

[Complete if necessary: Discussion points to OLP and imagination research etc.]

- Does this explanation make sense to you?
- What about the explanation made sense to you, what didn't?

Exercise of intake

We practiced the imaginary pill intake together during the intervention appointment.

- How was this exercise for you?
- Was this exercise helpful for the intake at home?
    - If so, why and how could it have been improved?
    - If not, why not and what would have helped you?

2. **Intervention phase** [Treatment during 3 weeks]

The next questions refer to the three week period before the exam during which you took the imaginary pills.

Pill intake

- Did you find it easy to take the imaginary pills? Were you able to access the image of the pill clearly? Tell me about it.
- Did the daily reminder emails help you to remember to take it as prescribed or would you have thought of taking it the anyway?
- Have you linked the intake with a ritual (e.g. breakfast in the morning)? If so with what was it?
- Was there a general feeling or a recurring thought when taking the pills? If so, what was it?
- Would you have wished that the imaginary pills were not to be taken at fixed times, but rather in situations where you would have needed them more (before learning or in case of increased anxiety)?

Effect of the pill

- Did you believe in the effects of the pill or were you skeptical about them?
- When you took the placebo, did you sometimes remember the explanation given during the intervention appointment? If so, what about it? If not, why not?
- During intake, did you think of the positive state you described back in the video call?
- Do you feel that the pills have helped you during the examination phase?
  - With regards to physical symptoms, thoughts or emotions?

Pill as such

- At the intervention appointment, you described your idea of the pill and the state it should bring you to *[i.e.: repeat pill characteristics and the feeling associated with them]* Did anything change in the effect or appearance of the pill when you took it at home?
- Is the pill the right shape or would you rather have imagined something else to get it in the desired state? (For example, a ritual like physical exercise?)

3. Exam situation

Effect of pill

- How did you feel during the exam?
- Do you think the pill helped in the exam situation?
  - With regards to physical symptoms, thoughts or emotions?

4. Future use

Assessment of effectiveness and future behaviour

With regards to a future use of imaginary pill:

- Would you use this method in a future situation where you need help (be it again before an exam or any other difficult situation)? If yes: Why? If not: Why not?
- Would you recommend this method to others? If so, to whom and why to this person?
