# Supplementary material for: A qualitative study of imaginary pills and open-label placebos in test anxiety
Source: PLoS One. 2023 Sep 1;18(9):e0291004. doi: 10.1371/journal.pone.0291004 (PMC10473493; doi:10.1371/journal.pone.0291004)
Supplement: S2 Appendix — (PDF) [file pone.0291004.s002.pdf]

S2 Appendix: Open-ended questions of RCT

Open-ended questions in open-label placebo group

1. Intervention Credibility: Did you find the explanation of why the placebo intervention can work helpful?
   - **a. Yes, why? (open-ended question)**
   - b. No, why? (open-ended question)
2. What do you think about the idea of taking placebo pills? (open-ended question)
3. Do you find the placebo pill has generally helped you to be less anxious/stressed before the exam? Yes/No
4. For which symptoms did the placebo pill help to which extent 0% (the pill did not help at all) - 100% (the pill helped 100%)
   - Concerning excitement (emotional and physical tension)
   - Concern (thoughts about failure, self-doubt)
   - Regarding distraction (distraction from the task by irrelevant thoughts)
   - Regarding confidence (self-worth)
5. **Did you assume that the placebo pills would work or were you skeptical? (open-ended question)**
6. What do you think was in the placebo pills ? (open-ended question)
7. What did you learn by participating in this treatment study ? (open-ended question)
8. Do you have any other comments ? (open-ended question)

Open-ended questions in imaginary pill group

1. Intervention Credibility: Did you find the explanation of why the placebo intervention can work helpful?
   - **a. Yes, why? (open-ended question)**
   - b. No, why? (open-ended question)
2. In general, how open are you to taking a pharmacological pill for your test anxiety ? 0% (not at all open) to 100% (very open) (slider).
3. Do you find the imaginary pill helped you to be less anxious/stressed before the exam ? Yes/No
4. For which symptoms did the imaginary pill help to what extent 0% (the pill did not help at all) - 100% (the pill helped 100%)
   - Regarding excitement (emotional and physical tension)
   - Regarding concerns (thoughts about failure, self-doubt)
   - Regarding distraction (distraction from the task by irrelevant thoughts)
   - Regarding confidence (self-worth)
5. How difficult was it for you to imagine the imaginary pill? 1 (very easy) - 7 (very difficult)
6. How well could you imagine the following aspects of the imaginary pill (0 - not at all well to 100 almost identical to a real pill):
   - Seeing the pill (visualization)
   - Tasting the pill
   - Feeling the pill
   - Effects of the pill

7. Did you find it easier to visualize and take the imaginary pill during the study?
   - Yes, it was easier
   - It did not change
   - No, it became more difficult
9. What do you think about the idea of taking an imaginary pill ? (open-ended question)
10. Did you assume that the imaginary pill would work or were you skeptical ? (open-ended question)
11. Did you learn anything from participating in this treatment study? If yes, what? (open-ended question)
12. Do you have any other comment? (open-ended question)
