# Supplementary material for: A qualitative study of imaginary pills and open-label placebos in test anxiety
Source: PLoS One. 2023 Sep 1;18(9):e0291004. doi: 10.1371/journal.pone.0291004 (PMC10473493; doi:10.1371/journal.pone.0291004)
Supplement: S1 File — (PDF) [file pone.0291004.s003.pdf]

S1-S3 Tables: Open-ended questions

S1 Table. Idea of the intervention. What do you think about the idea of taking an imaginary pill / placebo pill?

	OLP N=59	IP N=55	Total N=114
The idea is…		N (%)	
… excellent	10 (16.9)	7 (12.7)	17 (14.9)
… good	29 (49.2)	27 (49.1)	56 (49.1)
… fair	11 (18.6)	11 (20)	22 (19.3)
… poor	9 (15.3)	10 (18.2)	19 (16.7)

Note. IP imaginary pill, *OLP* open-label placebo

S2 Table. Credibility of explanation. How credible did you find the explanation of why an imaginary pill / open label placebo can work?

	Total N=114
The explanation was…	N (%)
… extremely credible	10 (8.77)
… very credible	63 (55.26)
… moderately credible	34 (29.82)
… hardly credible	2 (1.75)
… minimally credible	4 (3.51)
… not credible at all	1 (0.88)
**Helpfulness of explanation** How helpful did you find the explanation of why the imaginary pill / open label placebo can work?	Total N=114
The explanation was…	N (%)
… extremely helpful	11 (9.65)
… very helpful	62 (54.39)
… moderately helpful	36 (31.58)
… hardly helpful	2 (1.75)
… minimally helpful	2 (1.75)
… not helpful at all	1 (0.88)

S3 Table. Learning during treatment. Did you learn anything from participating in this treatment study? If yes, what?

	OLP N=65	IP N=66	Total N=131
I learned…		N (%)	
… how powerful our psyche/ imagination can be	8 (12.3)	12 (18.2)	20 (15.3)
… that mindfulness can be very helpful	10 (15.4)	7 (10.6)	17 (13)
… how to deal with anxiety	4 (6.2)	6 (9.1)	10 (7.6)
… to have more self confidence	5 (7.7)	4 (6.1)	9 (6.9)

… that IPs can actually work and be used daily	0 (0)	9 (13.6)	9 (6.9)
… that OLPs can actually work	8 (12.3)	0 (0)	8 (6.1)
… more about the placebo mechanism	7 (10.8)	0 (0)	7 (5.3)
… that daily routines help to clear thoughts	2 (3.1)	4 (6.1)	6 (4.6)
… that adherence is difficult	3 (4.6)	2 (3)	5 (3.8)
… how important expectations are	3 (4.6)	1 (1.5)	4 (3.1)
… something about myself	1 (1.5)	2 (3)	3 (2.3)
… a potentially new therapy method	0 (0)	2 (3)	2 (1.5)
… to always stay open minded	0 (0)	1 (1.5)	1 (0.8)
…to question conventional methods	0 (0)	1 (1.5)	1 (0.8)
… no	11 (16.9)	12 (18.2)	23 (17.6)
N/A	3 (4.6)	3 (4.5)	6 (4.6)
