# Supplementary material for: A qualitative study of imaginary pills and open-label placebos in test anxiety
Source: PLoS One. 2023 Sep 1;18(9):e0291004. doi: 10.1371/journal.pone.0291004 (PMC10473493; doi:10.1371/journal.pone.0291004)
Supplement: S2 File — (PDF) [file pone.0291004.s004.pdf]

S4 Table: Data imaginary pill characteristics

Aussehen							Effekte		
**Art** der Pille	**Form** der Pille	**Farbe** der Pille	**Verpackungs-form** der Pille	**Verpackungs - farbe** der Pille	**Grösse** der Pille	Weitere **Eigenschaften** der Pille	**Positiver Zustand** (welcher durch die Pille erreicht wird)	Weitere **positive Effekte**	**Nebeneffekte**
Tablette	länglich	weiss	viereckig	blau, rot, weiss	klein	-	Nimmt Nervosität, macht weniger zappelig, lässt mich mehr fokusieren, nimmt murmeliges Gefühl	-	Bisschen komisches Gefühl im Hals (so als konnte die Pille nicht ganz runtergeschluckt werden)
Tablette	rund	weiss	Blister	silber	klein	Wirkungen stehen auf der Verpackung	Senkung Puls, beruhigender, klarer Kopf, Selbstvertrauen	Konzentration/Fokus	trockener Mund, schwere Arme und Beine
Tablette	oval	weiss	Kartonschachtel und Blister	-	mittel	Gravur	durchbricht Gedankenkreise an die Prüfung	Linderung Darmbeschwerden	-
Kapsel	oval	pink weiss	Blister	-	mittel	mittelteuer, schmeckt leicht süsslich	Positiver Stress, Ruhegefühl mit einer nötigen Menge an Nervosität für die Konzentration	beruhigend (Puls senkend, Zittern entgegenwirkend), Selbstvertrauen schenkend, Objektivität	ein leichtes "ameiseln" in den Fingerspitzen (nur kurz)
Kapsel	oval (abgerundeter Zylinder)	Sonnen-gelb weiss	Metalldose	-	klein	harte trockene Konsistenz, klebt auf Zunge , Kügelchen innerhalb Pille, die rascheln	ruhig, bewusst, sachlich, analytisch, geordnete/strukturierte Gedanken, Klarheit, zentriert/in mir ruhend, aktionsbereit, Bewältigungsgefühl, Konzentration, Selbstsicherheit und Vertrauen	leichtes Lächeln, langsame Atmung, langsame Herzfrequenz, Muskeln nicht zittrig/angespannt, aber einsatzklar	Ruhe, Gelassenheit, Entspannung (nicht zu viel)
Tablette	rund	weiss	Blister	-	mittel	glatte Oberfläche	Konzentration	Motivation und Beruhigung	-
Tablette	rund	pink	Blister	-	klein	-	Gelassenheit, sich entspannt fühlen	negative Gedanken blockieren	versprüht Wärme
Tablette	rund	blau	Blister	-	klein	-	fokussiert, entspannt, optimistisch, motiviert	-	angenehm warmes und frisches Gefühl
Tablette	rund	hellgelb	Becher mit Deckel	-	klein	neutraler Geruch/Geschmack	körperliche Entspannung, Steigerung Aufmerksamkeit	Gefühl von Wärme (aus dem Bauch verbreitend)	-
Tablette	rund und kantig	weiss	Glasbehälter	braun	sehr klein	keine Nebenwirkungen, pflanzlich	fördert Hirndurchblutung, ermöglicht schnelleres Denken und Reagieren	-	-
Tablette	länglich	blau	Pillendose	braun mit weissem Deckel	mittel	ähnlich wie Schmerztablette	Beruhigung, Geborgenheit, Zuversicht, Selbstbewusstsein	Gefühl der sozialen Unterstützung, Fokus auf wichtige Dinge (Ausblenden von irrelevanten)	verringerter Puls, weniger "Zappeln", "schwerer" Körper
Tablette	rund	blau	Blister	-	mittel	glatte Oberfläche	Situationskontrolle, konzentriertheit/Fokussiertheit, Kompetenz, Selbstwirksamkeit, Wissen über eigene Fähigkeiten/Können	Selbstsicherheit, Vertrauen in eigene/s Fähigkeiten/Wissen	Stärkegefühl, selbstbewusst, weiss was ich tue, kann meine Stärken einbringen
Tablette	rund	weiss	Karton	grün/weiss	klein	-	ruhiger konzentrierter Zustand, kein Händezittern, weniger Druckgefühl, bzw. nur so ein Druckgefühl, dass es hilfreich ist	-	leichte Schläfrigkeit

Dragee	oval	neonblau (transparent)	Stoffsäcklein	-	gross	zergeht auf der Zunge (ähnlich wie ein Bonbon)	Leere im Kopf, Leichtigkeit in den Extremitäten, leichte Euphorie, Brustbereich wird nach oben (in die Luft) gezogen, Selbstvertrauen	Angenehme Körpertemperatur, verstärkt Sinneswahrnehmung	Wärmegefühl im Magen/Bauchbereich beim come up
Tablette	länglich	weiss	länglich	weiss mit grünen Streifen	mittel	glatte Oberfläche	ruhiger Effekt, gelassen, Fokus auf den Lernstoff	-	evtl schwache Übelkeit/Schwindel
Tablette	rund bis oval	pink	-	weiss mit pinker Schrift	mittel	Pille hat Plastik-Ummantelung, die man aber als solche einnehmen kann	narkotisierend, beruhigend, sie fokussiert, stoppt das Schwitzen	Wahrnehmung der Pille als zuverlässig	schwerer Kopf, kurzanhaltende Müdigkeit, Kribbeln
Tablette	rund	rosa	Glasbehälter	-	klein	einzeln verpackt, mittlerer Preis, lokal produziert (nicht durch Pharma)	Konzentration, Klar- und Wachheit, Gefühl der unangestrengten Kontrolle	angenehme und positive Grundstimmung, zuversichtlich aber nicht euphorisch	-
Tablette	kleine Smarties	grün	Kartonschachtel und Blister	-	sehr klein	sieht aus wie kleine Smarties, leicht zu schlucken	mehr Konzerntration/Fokus, mehr Selbstvertrauen und entspannterer Körper	kühlende Wirkung, Puls weniger stark spürbar	leicht trockener Mund
Tablette	rund	gelb	Blister	-	klein	-	Entspannung, Ruhe, innere Wärme	Fokus und Aufmerksamkeit der Gedanken	-
Lutschtablette	rund bis oval	rötlich	Aluminiumdose	-	mittel	süsslicher Geschmack	körperliche Gelassenheit aber gleichzeitig Fokus auf Inhalt und Prüfungs-Wissen	Selbstvertrauen und Sicherheit während der Prüfung	angenehme körperliche Schwerheit
Kapsel	oval	dunkelblau / hellblau	Kartonschachtel und Blister	-	klein	Pille schmeckt bitter und ein wenig unangenehm	warmes Gefühl von Körpermitte heraus, Entspannung, angenehme Schwere	Selbstbewusstsein steigt, Fokus geschärft, man spürt viel Energie	-
Tablette	rund	weiss	-	rot	klein	-	entspannt, nimmt Nervosität weg, führt zu mehr Selbstsicherheit	-	-
Tablette	rund	gelb	Runde Glasdose mit Klickverschluss	braun	klein	glatt	aufgeregte positive Anspannung bzw. auch Entspannung, kribbeln im Körper bzw. auf der Haut	man fühlt sich einfach gut danach	trockener Mund, die Arme fühlen sich so ein bisschen schwer an
Tablette	rund	weiss	Schachtel mit Streifen	-	klein	überzogen mit einem süsslichen Überzug	Entspannung, Beruhigung	keine Übelkeit	evtl Müdigkeit
Tablette	elliptisch	dunkelgelb	Blister	-	mittelgross	Grösse von einem Kaugummi	Wärme, Gelassenheit	niedriger Puls	schweissige Hände
Kapsel	kapselförmig	hellblau	Blister	-	mittelgross	geschmacksneutral	Innere Ruhe	Entspanung, Gefühl der Leichtigkeit	angenehmes Kribbeln im Bauch
Tablette	rund	weiss	Glasbehälter	-	klein	-	Beruhigung des Herzschlags und der zitternden Hände, klarer Kopf, allgemein ruhig	-	bisschen trockener Mund
Tablette	rund	grün	kleines Konfiglas	-	klein	leuchtet leicht	Leichtigkeit körperlich sowie auch gedanklich, beruhigt, macht einen glücklich und das Gefühl von Freiheit kommt auf, Fokus	lässt einen tief durchatmen, mentholartiger Geschmack	Gefühl von Kälte im Magen, Schwindelgefühl
Kapsel	oval	blau / weiss	kleines Glas	-	mittel	-	Gelassenheit, Entspannung, glücklich	-	Kopfschmerzen, Ausschlag
Tablette	oval	weiss	Alu	-	mittel	muss man mit Wasser einnehmen	Beruhigung, mehr Konzentration, weniger Ängste	-	-

Tablette	rund	hellgelb	Kartonschachtel und Blister	-	klein	etwas bitter	Energie, Fokus, Klarheit	angenehme Wärme	spätere Erschöpfung
Kapsel	kapselförmig	weiss blau	Dose	-	mittel	-	Ruhe, langsamer Herzschlag, ruhige Atmung	Gelassenheit, Wissen	Hunger
Tablette	rund	blau	Alu und Plastik zum rausdrücken	-	klein	schmeckt süss	innere Ruhe, Konzentration, Motivation produktiv zu sein	-	einem wird warm, trockener Mund
Kapsel	länglich	grau	sehr minimalistisch	weiss	mittel	bitter	Fokussiert, macht generell ruhiger, unterdrückt/schwächt andere evtl. negative Gedanken bzgl. Der Prüfung , macht gedanklich strukturierter/klarer	-	-
Kapsel	oval	weiss rosa	Blister	-	klein	Gelatine Hülle	Fokussiertheit und Ruhe	Lösung von Spannung	etwas heiss am Anfang
Tablette	länglich	türkis	Rationierungsdose	-	gross/sehr gross	Bitterer Geschmack auf der Zunge	heitere Stimmung, fokussiert und geerdet, ruhig und trotzdem wach, selbstbewusst	gesenkter Herzschlag	Gefühl von Wärme
Kapsel	rund	hellblau	Glasbehälter	braun	klein	glänzend	entspannend, ruhige Hände, beruhigend, guter Schlaf, schafft klaren Kopf	Verdauung, Bauchgegend schonend und beruhigend	trockener Mund, schwere Gliedmassen, heisser Kopf
Tablette	rund	weiss	Glasbehälter	-	mittel	-	innere Ruhe, Entspannung, Reduktion von Nervosität	-	trockener Mund
Tablette	oval	lavendel	Blister	-	mittel		Glücksgefühle	bekämpft Müdigkeit	-
Tablette	rund	weiss	Blister	-	mittel	hart und rau	Schleier der Angst geht weg, Freude und Motivation kommt	Ankommen bei mir, im hier und jetzt, Gefühl des im-Fluss-seins	Muskelrelaxion, Gefühl der Verbundenheut, Gelassenheit, Liebesgefühl
Tablette	rund	weiss	Kleines Glas mit Aludeckel und Etikette	-	klein	-	Entspannung der inneren Spannungszustände und der gesamten Muskulatur, bessere Konzentrationsfäähigkeit, bessere Stimmung, lässt Sorgen vergessen	Wärmegefühl vom Bauch ausgehend	-
Kapsel	kapselförmig	weiss mit roter Ummantelung in der Mitte	Dose mit weissem Deckel	-	mittel	Gelatineartige Ummantelung	Bauchkrämpfe verschwinden, ruhiges Atmen, Konzentration und Motivation steigt an, wacher	-	seitliches Stechen im Kopf, Oberkörper wird warm und die Hände schwitzig, Arme werden schwerer
Tablette	rund (fizzers-form)	weiss	Plastikdose mit Drehdeckel	-	klein	-	gutes warmes Gefühl, Gefühl der Sicherheit, krampflösend, soll Übelkeit verhindern	-	-
Tablette	oval	weiss	graue Verpackung zum Herausdrücken	-	mittel	-	bessere Konzentration	Senkung Blutdruck	Hitze, Schwitzen
Tablette	rund	weiss	Blister	-	klein	kein Geschmack	Entspannung	innere Ruhe	Schläfrigkeit
Kapsel	ründlich	rot	durchsichtig	-	klein	leuchtend	passt Arousal an (in beide Richtungen), führt zu besserem Fokus	-	-
Tablette	rund	weiss	Blister mit Firma/Wirkstoff	blaue Streifen	klein	Spalt in der Mitte	Entspanntheit, Kopf frei, Gefühl des losgelöst-sein, Flow, Selbstbewusstsein "Ich kann das"	Konzentrationssteigerung durch Interesse und Selbstbewusstsein	-
Kapsel	kapselförmig, rundliche Enden	violett	Kartonschachtel und Blister	-	klein	-	Selbstsicherheit, Selbstvertrauen, positive Stimmung	kongitive Kontrolle, Entspannung aber gleichzeitig auch eine Aktivität des Körpers	-
Kapsel	kapselförmig	rot weiss	Kartonschachtel und Blister	weiss	mittel	undurchsichtig	beruhigend, macht zuversichtlich und konzentrationsfördernd	gibt einen Energy-Boost	leichte Übelkeit
Tablette	rund	blau	Blister	-	sehr klein	-	macht ruhiger, weniger negative Gedanken, besserer Schlaf	Gefühl von Ruhe und Wohlsein, wie beim Singen	Wärme im Körper

Tablette	rund	dunkelgrün		Glasbehälter mit Metalldrehverschluss	-	mittel	auf Etikett ist eine einzelne schwarze Musiknote	Selbstbewusstsein, Kontrolle der Gedanken und Gefühle, Leichtigkeit	alles fliesst, Naturverbundenheit	Mundtrockenheit
Dragee	kugelrund	grün		keine	-	gross	-	kein Gedankenkreisen, im Moment sein, Fokus	Glückshormone	Zu stark fokussiert sein, dass man seine Umgebung nicht mehr so gut wahrnimmt
Tablette	rund	weiss		viereckig	-	klein	hat einen Strich in der Mitte	Förderung der Konzentration	Stressreduktion	Kopfschmerzen
Tablette	und	weiss		Normale, seriös wirkende Medikamentenverpackung	-	klein	kompakt, sollte als Ganzes geschluckt werden, ist nicht zum Zerbeissen/Zergehen lassen	beruhigend, konzentrationsfördernd, Puls wird tiefer, Atmung wird langsamer/tiefer/unruhiger, Wach und fit	Führt zu warmen, wohlige Gefühl im Bauch	Mundtrockenheit, wodurch es hilfreich ist, Wasser zu trinken
Dragee	rund	weiss		rundes Döschen aus Gold	-	sehr klein	hat raue Oberfläche, Dose macht ein Ton beim Öffnen	wohliges Gefühl, Wärme, der Hintergrund verschwimmt/wird leise, Ruhe, die ganze Energie kommt in den Kopf für die Konzentration	-	leise Musik spielt sich im Hintergrund ab
Tablette	rund	blau	weiss	Kartonschachtel und Blister	-	klein	-	beruhigend, Puls senkend, nervöses Zucken der Beine wird unterdrückt	unterdrückt allenfalls vorhandene sublimale Blockade	trockener Mund, stärkere Farbwahrnehmung
Bonbon	rund mit Zacken (wie kleine Sternchen)	gelb, pink	blau, grün	durchsichtiges Döschen mit Holzdeckel	-	klein	-	Entspannung, bringt positive Gedanken hervor	-	Müdigkeit

Anmerkung. Tabelle beinhaltet auch Pillencharakteristiken von Personen die für die finale Analyse nicht verwendet wurden (da sie ausgeschlossen wurden)
