# Supplementary material for: A qualitative study of imaginary pills and open-label placebos in test anxiety
Source: PLoS One. 2023 Sep 1;18(9):e0291004. doi: 10.1371/journal.pone.0291004 (PMC10473493; doi:10.1371/journal.pone.0291004)
Supplement: S3 File — (PDF) [file pone.0291004.s005.pdf]

S5-S9 Tables: Data from open-ended questions from RCT.

Table S5. Helpfulness of explanation. Why did you find the explanation that the imaginary pill / open-label placebo helpful?

group	helpfulness (Warum fanden Sie die Erklärung zu IP / OLP hilfreich?)	helpfulness of explanation
OLP	Weil mir **bisher nur schwammig** klar war, wie eine Tablette ohne Wirkstoff wirken soll.	explanation gave new knowledge, explanation led to better understanding
OLP	weil man es sich **besser vorstellen** konnte so	explanation made imagination easier
OLP	Habe [eine Arbeit] über den Placeboeffekt geschrieben und es war darum spannend an einer Open-Labelstudie mitzumachen. Die Erklärungen waren mir bereits bekannt, jedoch fand ich es trotzdem im Rahmen der Studie hilfreich, weil es **auf die Prüfungsangst bezogen** war.	explanation strengthened previous knowledge/ beliefs, explanation led to better understanding
OLP	Weil die Placeboeffekte einem **nicht immer gleich bewusst** sind und sie **oft gar nicht erwähnt** werden.	explanation gave new knowledge
OLP	Ich konnte mir **vorstellen**, dass Placebotablette schlucken, begleitende konditionieren Prozesse im Gehirn auslösen und steuern könnten.	explanation led to better understanding, explanation made imagination easier
OLP	Es wurde eine **Studie erwähnt** in Deutschland, die bereits durchgeführt wurde Ich kenne mich etwas mit offenen Placebos aus, und war deshalb neugierig ob es wirklich wirkt	mentioning previous studies
OLP	weil die **positiven aspekte** der behandlung hervorgehoben werden	explanation focused on positive aspects
OLP	Weil es die Möglichkeit einer Wirkung **offen lässt**.	left an open outcome
OLP	eigene **Erwartung** auf Besserung schafft **Zuversicht**, diese wiederum verbessert das allgemeine Befinden<br>2x täglich **aktiv** dran zu glauben, dass durch die Einnahme die Prüfungsangst besser wird und aktiv etwas dafür zu tun, dass es besser wird, hat meine Selbstwirksamkeit verstärkt	explanation created expectation, explanation created faith
OLP	Ich fand die **Erklärung hilfreich**, weiss jetzt aber ehrlich gesagt nicht mehr, was die Erklärung war.	explanation was helpful
OLP	**Tricks** the body and mind into thinking the drug can work well for a certain condition	explanation created expectation
OLP	Weil eine **Erwartungshaltung** das Empfinden stark beeinflussen kann	explanation created expectation
OLP	Es macht **Sinn**, dass man die positiven Gefühle verstärken kann, indem man sie sich regelmässig ins Gedächtnis ruft und sich daran erinnert.	explanation made sense
OLP	Hat bereits **Studie** gegeben, die das aufgezeigt hat. Aber auch, weil es mir plausibel erscheint, dass man durch den **Lerneffekt** "lernt".	mentioning previous studies, explanation was believable, explanation led to better understanding
OLP	weil es einem hilft den Prozess **besser nachzuvollziehen**	explanation led to better understanding
OLP	Normalerweise werden Placebopillen als "leer" bezeichnet und als ob sie überhaupt nichts bringen würden (quasi nur Platzhalter sind). Eine **richtige Erklärung** zu bekommen hat geholfen, da ich so auch selber daran glauben kann, dass das Placebo etwas bringt/eine Wirkung hat.	explanation led to better understanding, explanation created faith
OLP	Mir hat der Gedanke geholfen, dass mir allein durch den Prozess der Einnahme (durch das Schlucken) der Pille geholfen wird und ich somit **nicht aktiv an die Wirksamkeit glauben muss**, um eine Wirkung zu erhalten.	explanation led to better understanding
OLP	Weil es einen mentalen Effekt erzeugt. Die Psyche kann meiner Meinung nach eine Auswirkung auf die Gesundheit und auf den Körper haben.	explanation strengthened previous knowledge/ beliefs
OLP	Weil ich dadurch mehr **Sinn** darin verstand, sie trotzdem zu nehmen, auch wenn kein Wirkstoff drin war.	explanation made sense
OLP	weil es **schlüssig** war und obwohl ich nie davon gehört hatte, dass placebos auch wirken können, wenn man weiß, dass es placebos sind, machte die **erklärung sinn**.	explanation was believable, explanation made sense
OLP	interessant, und wenn man eine **Erklärung** hat, kann man sich vieles **besser vorstellen**.	explanation led to better understanding
OLP	Weil mir bewusst ist wie viel unsere **Psyche mitbeeinflussen** kann	improved mindfulness
OLP	Das Vorgehen wurde [hilfreich] **erklärt**	explanation was helpful
OLP	Weil sie die **Glaubwürdigkeit** erhöht hat.	explanation was credible
OLP	Es ist interessant zu wissen, aus welchen Gründen man die Tabletten nehmen soll und man kann sich besser **einreden**, dass es funktioniert, wenn man erzählt bekommen hat, **dass es bei anderen funktioniert hat**.	explanation led to better understanding, explanation created faith, mentioning previos studies
OLP	Sie wurde **ausführlich erklärt**	explanation was believable
OLP	Es hat sich auf **rationale psychologische Phänomene** basiert und deshalb fand ich es glaubwürdig	explanation was credible
OLP	Ja, dann habe ich einen **Sinn** darin gesehen, die Tabletten zu nehmen.	explanation made sense
OLP	Dadurch habe ich mehr daran **geglaubt**, dass sie auch wirklich etwas bewirkt.	explanation created faith
OLP	Damit man eine **erklarung** hat	explanation was helpful
OLP	Ich konnte mir so ein **besseres Bild** machen von der Studie.	explanation made imagination easier

OLP	Weil ich daran **glaube**, dass alles psychisch ist	explanation strengthened previous knowledge/ beliefs, explanation created faith
OLP	Erneut gehört, **wieso** sie wirken => hat mich in meiner Meinung **bestärkt**	explanation strengthened previous knowledge/ beliefs
OLP	Zu wissen, dass es schon **Studien** gibt, die **belegen** können dass und wie die Pillen wirken, hat mein **Vertrauen** darin bestärkt.	mentioning previos studies, explanation created faith
OLP	N/A	N/A
OLP	Weil ich so mit einem **positiverem Gefühl** in die Studie gegangen bin.	explanation focused on positive aspects
OLP	Ja, weil die Erklärung **Sinn** gemacht hat.	explanation made sense
OLP	Ich hatte es selber schon im studium	explanation strengthened previous knowledge/ beliefs
OLP	Ja, sie hat mir geholfen zu verstehen, dass **teilweise alles Kopfsache** ist.	improved mindfulness
OLP	Weil es für mich **Sinn** macht, dass auch der Körper darauf reagiert an was man selbst **glaubt**.	explanation made sense, explanation created faith
OLP	Um einen "Wirkmechanismus" zu erzielen	explanation led to better understanding
OLP	Weil man besser **aufgeklärt** ist.	explanation gave new knowledge
OLP	Mit der Erklärung konnte mich mehr **darauf einlassen** und somit auch an den Nutzen des Placebos **glauben**, was wahrscheinlich sehr geholfen hat...	explanation was helpful, explanation created faith
OLP	**Ansonsten gar nicht vorstellbar** warum mir es überhaupt etwas bringen sollte	explanation made imagination easier
OLP	Weil es teilweise dazu führte an die Wirkung zu **glauben**.	explanation created faith
OLP	Ja, weil somit die Versuchspersonen **bewusst** sein könnten	improved mindfulness
OLP	Es **stimmt** mit meinem Glauben **überein**	explanation strengthened previous knowledge/ beliefs
OLP	Weil sie mir durch das **neue Wissen Sicherheit** gegeben hat.	explanation gave new knowledge
OLP	Weil ich denke das unsere Psyche sehr grossen **Einfluss** auf unser Leben hat & die Placebopillen uns **mental stärken können**.	explanation strengthened previous knowledge/ beliefs
OLP	Weil ich es **interessant** finde, dass man auch eine Wirkung ohne pharmakologischen Wirkstoff erzielen kann.	explanation was interesting, conforming to personal interest
OLP	Um daran zu **glauben**.	explanation created faith
OLP	Um sich so **besser vorzustellen** auf was man sich einlassen kann und soll.	explanation made imagination easier
OLP	Sie klingt sehr **plausibel** und ergibt **Sinn**.	explanation made sense (2x)
OLP	Ich hatte vorher **noch nie davon gehört**, dass auch eine offene Verabreichung von Placebos wirken kann, daher fand ich dies sehr spannend	explanation gave new knowledge
OLP	Die **Erklärung**, dass der Körper Hormone ausschüttet bevor die wirkung eintritt weil man als Kind die positive Wirkung von Medikamenten erlernt hat.	explanation led to better understanding
OLP	War **aufschlussreich**	explanation gave new knowledge, explanation was helpful
OLP	Weil **Studien** den Effekt belegen. Wenn dies nicht so erklärt worden, wäre, hätte ich dies nicht geglaubt	mentioning previos studies, explanation led to better understanding
OLP	Ich **wusste zuvor nichts** über offene placebos.	explanation gave new knowledge
OLP	**Ohne** solch eine **Erklärung** hätte ich wahrscheinlich **gar kein Effekt** erlebt, da ich skeptischer gewesen wäre und quasi mich dagegen Wehren würde (glaube ich zumindest).	explanation gave security, explanation created expectation, explanation led to better understanding
IP	Eine **gute Erklärung** erhöht für mich die Glaubwürdigkeit.	explanation made sense, explanation was believable
IP	Weil ich an den Effekt des Placebos **glaube**. Und ich war gespannt, wie es sich bei mir auswirken wird. Die Erklärung schien mir **plausibel**, weil sie gut begründet wurde. Ich kann leider nicht mehr genau sagen, was dass ich an der Erklärung am besten gefunden habe, aber ich war auf jeden Fall **überzeugt davon**	explanation made sense, explanation strengthened previous knowledge/ beliefs, explanation created faith
IP	Weil ich an die Selbstheilungskräfte des Körpers **glaube**, und es auch Phänomene gibt, die nur so erklärt werden können. Ich **glaube** daran, dass man mit den Gedanken alleine sehr viel bewirken kann, und somit auch mit einer imaginären Pille.	explanation strengthened previous knowledge/ beliefs, explanation made sense, explanation improved mindfulness
IP	Die **Erklärung** hat mir geholfen mehr an die imaginäre Pille zu **glauben**.	explanation created faith
IP	Ich denke die Pille hat mir ein Erinnerungsanstoss gegeben, was ich mit ihr **erreichen möchte** und dadurch habe ich mir selber gesagt: 'du kannst das'.	explanation created expectations
IP	Die Verbindung von **Placebo Effekten (als Erwartungs-und Lerneffekte) und Mental Imagery** macht für mich **Sinn** und ist eine interessante Verknüpfung. Das "Einnehmen" hat für mich dann geholfen, wenn ich es mir tatsächlich möglichst bildlich vorgestellt habe und auch die körperliche Wirkung möglichst echt vorgestellt habe.	explanation made imagination easier, explanation led to better understanding
IP	Weil die **Erklärung** es mir ermöglichte offen dem Placebo gegenüberzustehen. Ich hatte **keine Vorurteile** dem Placebo gegenüber.So konnte ich ganz neutral, mir täglich mehrere Minuten Zeit nehmen, mich gedanklich mit der Prüfung konfrontieren.	explanation left an open outcome

IP	Damit ich **glaub** kann, dass es schon mal gewirkt hat.	explanation created faith
IP	Wenn man ein **Gefühl** der Entspannung durch die Einnahme der imaginäre Pille **erzeugen** kann, fühlt man sich mehr entspannt und wird dann dadurch auch mehr entspannt	explanation improved mindfulness
IP	N/A	N/A
IP	da es bereits **studien** gibt, die die wirkung bestätigt haben.	mentioning previos studies
IP	Gedanken und Vorstellungen können unser Handeln beeinflussen. Deshalb hat es in meinen Augen **Sinn** gemacht, dass alleine die Vorstellung eine Pille zu nehmen (der Vorgang der Einnahme und der anschließenden Wirkung im Körper) bereits etwas bei manchen Personen bewirken könnte. Im Prinzip ist das **nicht weit entfernt von einer Placebo Pille**, außer dass man sich die Einnahme nur vorstellt und nicht wirklich durchführt. Ich glaube aber schon, dass das in der Stärke der Wirkung einen Unterschied macht.	explanation led to better understanding, explanation strengthened previous knowledge/ beliefs, explanation made sense
IP	finde ich schwierig zu beantowrten aber prinzipiell macht es für mich **sinn**	explanation made sense
IP	Um die **Erwartung** zu steigern.	explanation created expectations
IP	Weil ich auch der Meinung bin, dass sich vieles im Kopf abspielt und nicht wirklich von Heilmitteln geholfen werden kann.	explanation strengthened previous knowledge/ beliefs
IP	Die Erklärung hat für mich **Sinn** ergeben. Als ich die Pille das erste Mal genommen habe, habe ich mich auch gestärkt gefühlt.	explanation made sense, explanation created faith
IP	N/A	N/A
IP	Weil ich das Konzept/ die Idee dahinter besser verstehen konnte und ich auch **überzeugter von der Intervention** war, als wenn ich keine Erklärung bekommen hätte.	explanation led to better understanding, explanation was helpful
IP	Dank der **Erklärung** hatte ich zu Beginn der Einnahme wirklich das Gefühl/die **Hoffnung**, dass es helfen wird	explanation created faith, participant could feel effect
IP	Weil man dadurch noch **mehr Vertrauen** in die Pille gesetzt hat.	explanation created faith
IP	N/A	N/A
IP	Die Erklärung, dass wenn man sich etwas bildlich vorstellt dieselbe Hirnareale aktiv werden, wie wenn man etwas sieht, wusste ich nicht, fand ich aber **nachvollziehbar** und deshalb konnte ich es mir dank der Erklärung **besser vorstellen** warum es wirkt.	explanation made sense, explanation gave new knowledge, explanation made imagination easier
IP	dadurch wurde es real, man konnte sich selbst praktisch vormachen warum das jetzt so passiert -> also **Logik** hilft	explanation made sense,
IP	So weiss ich dass sie auch **bei anderen Personen** wirken kann bzw **schon gewirkt** hat	mentioning previos studies
IP	Da der Mensch durch seine eigene **Überzeugungskraft** im Leben voran kommt und man so automatisch nach dem Ziel strebt.	explanation strengthened previous knowledge/ beliefs
IP	Es war **nachvollziehbar**, dass ein imaginäres Placebo wirken kann, wenn ein echtes Placebo auch aufgrund der **Erwartungen** des Probanden wirkt.	explanation made sense
IP	Die **Erklärung** half mir mich selbst von möglichen Wirkungen der Studie zu **überzeugen**	explanation strengthened previous knowledge/ beliefs
IP	durch die **überzeugte** haltung der forscherin, klang die erklärung sehr **plausibel** für mich	explanation made sense, showed researcher allegiance
IP	**einfacher zum vorstellen**	explanation made imagination easier
IP	Ich wusste nicht, dass **andere Studien** dies schon durchgeführt haben mit Erfolg	mentioning previous studies
IP	Weil ich **vorher noch nie davon gehört** habe und ich mir gut vorstellen kann, dass wenn man dies über eine längere Zeit durchführt, es positive Ergebnisse bewirken kann.	explanation gave new knowledge, explanation improved mindfulness
IP	**Mechanismus verstehen** der abläuft	explanation led to better understanding
IP	Die Erklärung ergab **Sinn**.	explanation made sense
IP	Ich kann daher **nachvollziehen**, warum es für manche Menschen wirken kann.	explanation made sense
IP	Weil ich mir alles **besser vorstellen** konnte	explanation made imagination easier
IP	wegen dem Placebo-Effekt	explanation was believable
IP	Weil man sich das selber nicht so genau überlegt. Es ist gut eine umfassende **Einführung** zu bekommen, wenn man an etwas glauben soll. Es wirkt dadurch auch **professioneller**, was den Effekt **verstärkt**, meiner Meinung nach. Hätte ich es nebenbei mal im Alltag gehört, hätte ich weniger daran geglaubt.	explanation made sense, explanation led to better understanding
IP	Die **Herleitung** aus open labe placebos mit fake Pillen war nachvollziehbar	explanation made sense
IP	Weil sie **wissenschaftlich fundiert** war.	mentioning previous studies
IP	wir hatten Placeboeffekte eben erst in der Vorlesung durchgenommen	explanation strengthened previous knowledge/ beliefs
IP	N/A	N/A
IP	N/A	N/A
IP	Weil man sich sonst nie Gedanken darüber macht, wie fest man sich **mental selbst unterstützen** kann. Diese **Erklärung** hat dies nochmals erneut aufgezeigt.	explanation improved mindfulness
IP	Es hat mir geholfen zu verstehen, **wie** diese Pillen **wirken**.	explanation led to better understanding
IP	Die **Erklärung** hat mir beim **Verständnis der Idee** einer imaginären Pille geholfen, folglich auch warum diese wirken kann.	explanation led to better understanding

IP	weil es sonst zu weit hergegriffen gewesen wäre... **ohne einführung** hätte ich es mir wahrscheinlich **nicht** so vorstellen können.	explanation led to better understanding, explanation gave new knowledge
IP	Weil praktisch alles mit der **mentalen Einstellung** zusammenhängt. Wenn diese stimmt & stabil ist, kann man so die eigene Leistung auch verbessern.	explanation strengthened previous knowledge/ beliefs, explanation improved mindfulness
IP	hat mehr **Sinn** gemacht, wieso ich sie nehme,obwohl sie eigentlich nicht existieren	explanation made sense
IP	N/A	N/A
IP	Ich kann mich nicht mehr gut an die Erklärung erinnern. Ich habe momentan nur mein eigens Wissen über Placebo im Kopf.	N/A
IP	Weil Placebos **bewiesen** sind.	explanation strengthened previous knowledge/ beliefs, mentioning previous studies
IP	Zu wissen, dass **offen verabreichter Placebo** bei vielen Menschen eine **Wirkung** erzielt, hat mir geholfen daran zu denken, dass auch die imaginäre Pille wirken könnte. Ausserdem habe ich gemerkt, dass es mir hilft, bei der Einnahme der Pille, die positiven Wirkungen der Pille ins Gedächtnis zu bringen und vorallem auch positive Gedanken bezüglich der Prüfung zu "lernen".	mentioning previos studies, explanation focused on positive aspects
IP	Durch die sich vorgestellte Wirkung kann man sich viel besser **auf sich selber konzentrieren** und auf seine Leistungen vertrauen.	explanation created faith, explanation improved mindfulness
IP	Damit die Einnahme dieser Pille auch **glaubwürdiger** war.	explanation created faith
IP	Die **Beispiele des Placeboeffekts waren beeindruckend** und haben den Gedankengang zum weitergeführten Gedanken der imaginären Pille erklärt.	mentioned previous studies, explanation led to better understanding

Table S6. Skepticism towards treatment. Did you assume that the imaginary pill / open-label placebo would work or were you skeptical?

group	skepticism (Sind Sie davon ausgegangen, dass die IP/OLP Intervention funktionieren würde, oder waren Sie skeptisch?)	skepticism
OLP	Ich war skeptisch aber gleichzeitig habe ich auch angenommen, dass sie wirken, weil sie eben auch dann wirken, wenn man weiss, dass es sich hierbei nur um Placebos handelt.	sceptical, believed in effect
OLP	ich war skeptisch aber erhoffte mir dennoch einen Effekt, also war nicht abgelehnt davon	sceptical, hopeful
OLP	Ich war skeptisch, sowieso hatte ich dieses mal fast keine Prüfungsangst weil ich sehr wenige Prüfungen hatte und alle wiederholt werden konnten. Darum empfand ich gar keinen grossen Anreiz meine Angst zu lindern.	sceptical
OLP	Ich war schon ein bisschen skeptisch. Habe aber trotzdem daran geglaubt.	sceptical, believed in effect
OLP	Ich habe mich darauf eingelassen, dass die Placebotabletten wirken.	open minded
OLP	Nein. Ich war nicht skeptisch. Wie gesagt, ich hatte schon vorher über offene Placebos gelesen und war neugierig.	believed in effect
OLP	skeptisch	sceptical
OLP	skeptisch aber offen	sceptical, open minded
OLP	ja, habe von Anfang an daran geglaubt, dass weil Ich etwas Konkretes gegen die Angst tue, dass diese besser wird	believed in effect
OLP	Ganz am Anfgang war ich skeptisch, habe mich aber darauf eingelassen (aber der ersten Pilleneinnahme)	sceptical, open minded
OLP	I wasn't sure	unsure
OLP	Ich dachte schon, dass sie wirken würde	believed in effect
OLP	Ich dachte mir, dass sie etwas wirken würden	believed in effect
OLP	Ich habe gewusst, dass sie Placebos wirken (theoretisch), war dennoch skeptisch, da ich es selber nie ausprobiert habe (praktisch).	sceptical
OLP	Ich war anfangs etwas skeptisch, aber ich habe schon bald deutliche Unterschiede zu anderen Prüfungsphasen gemerkt.	sceptical at first
OLP	Anfangs war ich nicht 100% überzeugt, aber ich war von der generellen Idee überzeugt also hat sich das nach einigen Tage gelegt. Und die regelmässigen Umfragen haben mir das selber auch gezeigt.	sceptical at first
OLP	Ich war skeptisch, da ich es schwer vorstellbar fand (und finde), dass etwas, von dem man weiss, dass es keinen Wirkstoff enthält, helfen kann. Ausserdem gehört die Prüfungsangst für mich in gewisser Weise zur Prüfung dazu, deshalb ich mir nicht wirklich vorstellen konnte, dass ich diese stark reduzieren könnte.	sceptical
OLP	Ich war eher skeptisch	sceptical
OLP	Ich wollte möglichst ohne Wertung die Studie durchführen, das heisst ich sagte mir „es kann gut sein, dass es hilft, jedoch kann es auch sein, dass sie nicht wirken“	open minded
OLP	ich war skeptisch, habe mir aber immer beim einnehmen vorgestellt, dass sie gegen Prüfungsangst helfen würde.	sceptical, open minded

OLP	Ich war skeptisch	sceptical
OLP	Ich bin von einer Wirkung ausgegangen	believed in effect
OLP	ich dachte, dass sie wirken werden	believed in effect
OLP	Ich war eher skeptisch.	sceptical
OLP	Ich war einerseits skeptisch, habe mir aber gewünscht und eingeredet, dass bestimmte Dinge durch die Pillen besser geworden sind.	sceptical, hopeful
OLP	Skeptisch	sceptical
OLP	Ich war sehr skeptisch	very sceptical
OLP	Ich war skeptisch	sceptical
OLP	Ich war offen dafür, dass sie etwas bewirken würden.	open minded
OLP	Skeptisch	sceptical
OLP	Ich hoffte, sie würden wirken.	hopeful
OLP	Ich habe daran geglaubt, dass sie wirken werden	believed in effect
OLP	Ich habe angenommen, dass sie wirken	believed in effect
OLP	Ich dachte schon, dass sie wirken würde, war aber dennoch überrascht dass sie so stark gewirkt hat.	believed in effect
OLP	Ich war skeptisch. Ich glaube, dass kann auch unter anderem ein Grund sein, dass sie nicht "gewirkt" haben.	sceptical
OLP	Ich war zuerst sehr skeptisch	sceptical
OLP	Ich war skeptisch	sceptical
OLP	Ja ich war etwas skeptisch	sceptical
OLP	Nein [skeptisch]	sceptical
OLP	Ich habe angenommen, dass sie wirken. Ein kleines bisschen war ich skeptisch.	believed in effect, slightly sceptical
OLP	Ich war skeptisch	sceptical
OLP	Ich war eigentlich neutral eingestellt, hätte aber vielleicht sogar "mehr" erwartet.	neutral
OLP	Ich war nicht skeptisch, mehr neugierig, was es verändern wird. In dem Sinne habe ich schon an den Nutzen geglaubt.	curious
OLP	Ich kannte die Wirkmechanismen, aber im Hinterkopf war immer der Gedanke, dass kein Wirkstoff enthalten ist.	open minded but sceptical
OLP	Ich habe schon gedacht, dass sie etwas wirken würde.	believed in effect
OLP	Ich war sehr überzeugt von die Wirkung der Placbopillen	believed in placebo effects
OLP	ich habe während der Einnahme sehr an die Wirkung der Placebos geglaubt	believed in placebo effects
OLP	Beides, aber mehrheitlich habe ich angenommen, dass sie wirken werden.	sceptical, believed in effect
OLP	Ich war relativ neutral eingestellt.	neutral
OLP	Ja, ich habe schon angenommen, dass sie wirken.	believed in effect
OLP	Ich war der Überzeugung, dass sie nützen werden.	believed in effect
OLP	Ich habe eine positive Einstellung gegenüber Placebos und habe eine eintretende Wirkung erwartet	believed in effects
OLP	Ich habe daran geglaubt, dass die Placebopille helfen wird.	believed in effects
OLP	Ich war eher skeptisch	sceptical
OLP	Ich war sehr skeptisch aber auch gespannt ob es trotzdem wirkt	sceptical
OLP	Ich habe daran geglaubt.	believed in effect
OLP	Ich war skeptisch. Ich hatte das Gefühl geframet zu werden während dem Gespräch.	sceptical
OLP	Ich habe gehofft, dass sie wirken, ich war aber auch skeptisch.	hopeful, sceptical
OLP	Ich war offen gegenüber den Placebopillen aber nach einer Zeit (ca.2 Wochen), wurde ich skeptischer.	open minded, grew more sceptical over time
IP	Ich war eher skeptisch zu Beginn.	sceptical at first
IP	Keines von beidem. Ich hab mich einfach mal darauf eingelassen. Nach dem Motto nützt es nichts, schadet es nicht.	not sceptical
IP	Ich habe definitiv von Anfang an an die Wirkung geglaubt, aber wusste nicht wie oder ob sie tatsächlich bei mit wirken würde.	believed in effect, sceptical
IP	Ich habe angenommen, dass die Pille wirken würde.	believed in effect
IP	ich habe angenommen sie wirkt	believed in effect
IP	Primär angenommen, dass es wirkt, auch wenn nicht unbedingt auf Grund der Vorstellung einer Pille selbst. Nicht skeptisch.	believed in effect, not sceptcical
IP	Ich machte mir dazu nicht viel Gedanken. Sicherlich war ich offen.	open minded
IP	Anfangs schon, aber es ist nicht viel passiert und deshalb wurde ich immer mehr skeptischer.	believed in effect but grew more sceptical over time
IP	Ich konnte mich schon vorstellen, dass die imaginäre PIlle wirken konnte um sich zu entspannen, aber nicht um die Prüfungsangst zu reduzieren (Erhöhung von Selbstwert, ...)	open minded, sceptical
IP	Ich war eher skeptisch und dachte nicht, dass es helfen würde.	slightly sceptical
IP	ich war skeptisch, habe mich aber so gut wie möglich darauf eingelassen	sceptical, open minded
IP	Ich war ehrlich gesagt etwas skeptisch. Ich habe keine allzu große bis gar keine Wirkung erwartet im Zusammenhang mit der Prüfungsangst. Ich könnte mir aber wie gesagt vorstellen, dass man in einer anderen Situation eine größere Wirkung erwarten könnte. Es braucht allerdings auch noch mehr Übung,	slightly sceptical

	damit man sich die Wirkung wirklich vorstellen kann. Das ist vermutlich einfacher, wenn es um eine physische Angelegenheit geht.	
IP	Skeptisch	sceptical
IP	War nicht skeptisch.	not sceptical
IP	Eigentlich hatte ich mir gedacht, es würde mir helfen, einfach die positiven Effekte dieser Pille immer wieder zu denken. Aber es hat nicht so funktioniert.	believed in effect
IP	Ich war zum Teil sehr zuversichtlich über die Wirkung der Pille. Auf der anderen Seite, wusste ich auch, dass ich mich oft aus der Ruhe bringen lasse, weswegen ich dann doch etwas skeptisch war, ob die Pille mich wirklich beruhigen würde.	sceptical
IP	eher skeptisch.	sceptical
IP	ich war sehr positiv eingestellt gegenüber der Studie, da bei mir auch der Wunsch nach einer "Verbesserung" da war.	open minded
IP	Ein bisschen von beidem und auch während der 3 Wochen hat sich meine Meinung immer wieder etwas geändert	believed in effect, sceptical
IP	Ich habe daran geglaubt, dass sie zumindest ein paar Effekte zeigen würde.	believed in effect
IP	ich war skeptisch	sceptical
IP	Ich war etwas skeptisch	slightly sceptical
IP	erst war ich skeptisch, aber von dem Gespräch per zoom wurde ich überzeugt	sceptical at first, believed in effect
IP	mittel. Ich weiss dass so was positive Effekte haben kann, dennoch war ich skeptisch	sceptical
IP	skeptisch.	sceptical
IP	Ja ich hatte an die Wirkung geglaubt und diese auch bereits bei der ersten "Einnahme" auf für mich beeindruckende Weise wahrgenommen.	believed in effect
IP	Ich dachte die Pille könnte Effekte hervorrufen, jedoch nicht in enormen Massen.	believed in effect
IP	ich war überzeugt, da ich bereits beim ersten erproben etwas wahrnehmen konnte	believed in effect
IP	Ich dachte, es wird helfen	believed in effect
IP	Ich bin sehr pro Vorstellung und Placebo und glaube an die Wirkung	believed in effect
IP	Ich wollte mich sicherlich darauf einlassen, aber war auch etwas skeptisch, ob das wirklich helfen kann.	open minded, sceptical
IP	Ich bin davon ausgegangen, dass sie wirken würde	believed in effect
IP	Ich war etwas skeptisch, aber habe es für möglich gehalten dass sie wirken könnte.	sceptical
IP	Ich war diesem Vorgang gegenüber eher skeptisch eingestellt.	sceptical
IP	Ich war relativ zuversichtlich, dass es irgendeine Art von positivem Effekt haben würde.	believed in effect
IP	Ich dachte schon das sie wirkt	believed in effect
IP	Ich war skeptisch aber habe auch schon viel über effektive Placebostudien gehört, was meine Skepsis vermindert hat.	sceptical
IP	Ich dachte schon, dass es einen Effekt geben wird	believed in effect
IP	Teils teils.	sceptical, believed in effect
IP	ich hatte grosse Hoffnung in die imaginäre Pille, aber nachdem ich nach der 1.Woche noch immer keine Wirkung merkte, wurde ich skeptisch.	hopeful, sceptical
IP	Ich war skeptisch.	sceptical
IP	Ich dachte sie wirkt, aber war mir nicht 100% sicher.	open minded, slightly sceptical
IP	Ich habe gehofft, dass sie wirkt. Ich war positiv eingestellt aber wollte mich einfach überraschen lassen.	hopeful, open minded
IP	Anfangs war ich etwas skeptisch. Nach der ersten Woche habe ich voll und ganz an sie und ihre Wirkung geglaubt.	sceptical, believed in effect
IP	Ich hatte effektiv das Gefühl, dass die Pille wirkt bis zum Prüfungsmoment, da ich noch nie so "entspannt" an die Prüfung gegangen bin.	believed in effect
IP	Ich war sehr skeptisch und dachte nicht, dass es wiklich wirkt. Da ich jedoch weiss, dass der Placeboeffekt tatsächlich wirkt, war mir trotzdem das Potenzial bewusst.	sceptical, open minded
IP	Anfangs war ich skeptisch, aber wie gesagt, hängt alles mit der mentalen Verfassung zusammen. Ich habe mir das also auch eingeredet, dass diese Pille mir jetzt helfen wird.	sceptical, believed in effect
IP	ich war ein bisschen skeptisch	slightly sceptical
IP	Ich war zuversichtlich, dass die Pille vielleicht wirken könnte und ich wollte auch, dass sie wirkt.	believed in effect but slightly sceptical
IP	Ja, ich war mir sicher dass die Pille kurzfrsitig wirken wird. Skeptisch war ich eben gegenüber der langfristigen (länger als 15min) Wirkung. Ob sie länger als 15 Minuten gewirkt hat, kann ich nicht beurteilen, da ich keine Kontrollgruppe habe.	sceptical, believed in effect
IP	Nein, ich habe versucht an die Wirkung zu glauben.	slightly sceptical
IP	Ich konnte mir gut vorstellen, dass sich eine Wirkung zeigen würde. Denn durch die Einnahme der Pille beschäftigt man sich mindestens zwei mal pro Tag konzentriert auf positive/gewünschte Wirkungen und Gedanken bezüglich der Stresssituation.	believed in effect
IP	Ja ich bin davon ausgegangen, dass sie mit helfen kann.	believed in effect
IP	Ich war eher skeptisch aber hatte durchaus ein Vetrauen, dass es sicher in irgend einer Weise hilfreich sein wird.	sceptical, believed in effect
IP	Skeptisch aber ich hatte angenommen es könnte einen kleinen Effekt haben.	sceptical, believed in effect

Table S7. Idea of the intervention. What do you think about the idea of taking an imaginary pill / placebo pill?

group	idea (Was halten Sie von der Idee, eine imaginäre Pille/ Placebopille zu nehmen?)	idea
OLP	Ich halte das für eine **gute** Idee, weil ja sogar nachgewiesen wurde, dass Placebopillen auch dann wirken, wenn man weiss, dass sie nur Placebos sind. Das heisst sie wirken so oder so.	good - they work
OLP	**Kann** sicher helfen, aber es ist **schwierig** wirklich die Etwartung aufzubauen, dass es helfen soll wenn man weiss, dass es dies "rein biologisch" gar nicht tut	fair - difficult to build expectation
OLP	Ich bin nach wie cor **skeptisch**, weil ich nicht weiss wie man die Adhärenz fördern kann bei Open-Label Placebostudien. Ich habe die Pille ebenfalls oftmals **vergessen** oder aus **Faulheit** nicht eingenommen.	poor - sceptic, need better adherence
OLP	Ich finde es eine **gute** Idee. Wahrscheinlich tun wir das auch oft ohne es zu wissen	good -
OLP	Ich glaube daran, dass ein Placebo **wirken kann**. Aber ich denke, dass in meinem Fall meine dysfunktionale kognitive Denkschematas trotzdem einen grösseren Einfluss auf meine Angst hatten. Ich hatte in den letzen Tagen immer wiederkehrende verunsichernde Gedanken. Und ich denke, dass diese **Gedanken stärker waren als das ‚Entgegenwirken' des Placebo**.	fair - potential, but dysfunctional thoughts are stronger
OLP	Ich finde die Idee **gut**. Ich kann mir aber vorstellen, das für mich nicht eine Pille, sondern eher etwas anderes wie Video schauen, ein Mantra sagen, Übungen machen oder solche Sachen eher überzeugen würde. Ich **nehme nicht so gerne Pillen, das assoziere ich immer mit Medikamenten.**	good - prefers other types of placebo, pill associated with drugs
OLP	**gut** bei kindern oder bei **leichten** medizinischen **beschwerden** (kopfschmerzen)	good - for light complaints
OLP	Grundsätzlich **spricht nichts dagegen** und es kann wahrscheinlich vielen helfen **ohne grosses Risiko**.	fair - won't hurt to try, no risks
OLP	Hilfreich, wenn der Patient aufgeklärt ist und **daran glaubt**, d.h. dem Verabreichenden **vertraut**	fair - if you believe it, if you trust the provider
OLP	Finde ich **gut**. Warum Medikamente nehmen, die **Nebenwirkungen** haben könnten & gegen die man resistent werden könnte.	good - better than medicine with side effects
OLP	Was open to it	fair
OLP	Gerade bei **leichten** Beschwerden oder in Situation wo man kein Risiko damit eingeht finde ich das eine gute Sache	good - for light complaints, no risks
OLP	Es ist eine **gute Alternative** zu echten Medikamenten	good - alternative for drugs
OLP	Finde ich **gut**. Hat mir denke ich persönlich doch geholfen nicht ganz so aufgeregt zu sein. Jeden Tag zweimal aktiv daran zu denken, dass ich es schaffen werde war bestimmt auch hilfreich.	good - mindfulness
OLP	Ich finde es **super**, da es, trotz **Abwesenheit** eines tatsächlichen Wirkstoffes, einem **hilft** und in diesem Fall die Prüfungsangst zu minimieren.	excellent - decrease symptoms without active ingredients
OLP	Ich finde es gut, da man **keine pharmakologischen Mittel** einnimmt, die auch manchmal schädlich sein können oder viele **Nebenwirkungen** haben.	good - decrease symptoms without active ingredients, no risk of side effects
OLP	Ich finde es grundsätzlich eine **gute** Idee, da es mir leichter fällt, etwas einzunehmen, **statt einfach nur an etwas zu glauben**. Ich habe so das Gefühl, in einer gewissen Weise aktiv gegen meine Angst, etc. vorzugehen und einen Teil meiner Angst loslassen zu können, wobei sicher auch die Routine der Einnahme hilft.	good - tangible pill makes it easier than just believing
OLP	Ich würde es nicht nochmals machen, weil ich **nicht gerne ohne guten Grund Tabletten** zu mir nehme. Ich wusste das es sich um Placebopillen handelte, dennoch hatte ich das Gefühl 2x am Tag eine Tablette zu schlucken zu müssen **nicht gern**.	poor - taking pills for no reason
OLP	Ich denke, dass es bei **einigen** sehr **hilfreich** sein kann. **Andere** sind vielleicht zu **negativ** eingestellt, da würde es dann nichts bringen.	fair - depends on person
OLP	persönlich würde ich l**ieber richtige medikamente** nehmen, bei denen ich auch eine tatsächliche wirkung spüre	poor - prefers real drugs, didn't help
OLP	Interessant	good - interesting
OLP	Ich finde es eine **tolle Alternative** zu "herkömmlichen" Medikamenten. (Zumal man weiss, dass auch bei solchen Plazebo effekte eine tragende Rolle spielen)	good - alternative for drugs
OLP	Super	excellent -
OLP	Kann **für bestimmte Zwecke** hilfreich sein.	fair - could help with certain things
OLP	Ich weiss nicht, inwiefern es sich lohnt sich von Placebopillen abhängig zu machen. Ich bin der Meinung, dass ich lieber eine **Prüfung schaffe, ohne Hilfe** von aussen, dann weiss ich, dass ich allein das Zeug dazu hatte. Ausserdem weiss ich aus meiner Erfahrung, dass ich mir Dinge meist schlimmer vorstelle, als sie dann sind. Die Vorbereitung auf die Prüfung hat mich hier auch mehr Nerven gekostet, als die eigentliche Prüfungssituation, daher kann ich nicht sicher sein, dass die Pillen mir geholfen haben.	poor - dysfunctional thoughts are stronger, addiction to placebo
OLP	**Gute** idee aber man braucht viel **disziplin**	good - requires discipline
OLP	Es ist eine **gute Idee**, aber im Moment habe ich das Gefühl dass es **nicht gewirkt** hat	good - didn't work

OLP	Eine Interessante Idee. Es **interessiert** mich, wie stark die eigene Psyche einem helfen kann, obwohl man weiss, dass es nur Placebotabletten sind.	good - interesting
OLP	Ich finde es eine **gute** Idee und würde es wieder machen.	good - would do it again
OLP	**Nocht** viel	poor - didn't help
OLP	Ich finde es eigentlich eine **gute** Sache für verschiedene Bereiche.	good - could help with many things
OLP	Ich finde es **sehr gut**, dass solche Scheinmedikamente positive Wirkungen haben können.	excellent - works
OLP	**sehr gute** Idee	excellent -
OLP	Finde ich einen **guten** Ansatz. Denke damit kann man in Zukunft viel erreichen.	good - potential
OLP	Eigentlich eine **gute** Sache, da es wirklich Evidenz dafür gibt, dass sie wirken und man kann da eigentlich nichts verlieren. Einen Versuch ist es Wert (aber **bei mir persönlich eher nicht**)	good - won't hurt to try, didn't help
OLP	Sehr **hilfreich** und unterstützend	excellent - supportive
OLP	Finde ich **gut** solange man weiss und sichergehen kann, dass wirklich nichts drinnen ist und man **keine Nebenwirkungen** davon hat. Es ist zB auf lange Sicht **besser** bei irgendwelchen Schmerzen Placebos zu nehmen **als Schmerzmittel** (wenn es denn Hilft).	good - as long as pill is truly "empty" and no side effects
OLP	Finde ich **gut**	good -
OLP	Eine **sehr gute** Idee.	excellent -
OLP	Ist auf jeden Fall ein **Versuch wert**!	good - won't hurt to try
OLP	**Begrenzt hilfreich**. Ich bin davon überzeugt, dass die Einnahme von Placebopillen durchaus eine Wirkung auf Stress bzw. Prüfungsangst erzielen kann. Allerdings gehe ich davon aus, dass die erwartete Wirkung **nicht bei allen Probanden** eintritt, eben weil es auch eine Kopfsache ist.	fair - depends on person
OLP	Spannendes Experiment, ich glaube aber, ich hätte noch mehr und intensiver d**aran "glauben" sollen und die Einnahme bewusster machen.**	poor - requires a lot of faith
OLP	Ich fand es eine **sehr gute** Idee. Allgemein finde ich das Konzept, Placebo zu nehmen um Stress bzw. Angst zu verringern sehr **interessant** und eine tolle **Möglichkeit** für die Zukunft.	excellent - potential
OLP	Gut wenn man wirklich Beschwerden hat, ansonsten bin ich **kein Fan vom Griff zur Pille**. Medikamente (seien es „nur" Placebos) sollten nicht zur Gewohnheit werden meiner Meinung nach.	good - taking pills for no reason, addiction to placebo
OLP	An sich ist es eine **gute Idee**, ist zwar **keine riesige Hilfe**, aber hilft irgendwie trotzdem das Mentale unter Kontrolle zu haben.	good - mixed feelings
OLP	Auch wenn nicht ethisch ist, denke ich es wäre **besser nicht zu wissen, dass es um placebo handelt**	poor - deception would be better
OLP	Ich finde es **je nach Umständen sehr hilfreich**, mit der Einnahme von Placebo Pillen die Selbstheilungskräfte zu fördern und denke, dass diese in manchen Fällen ein zugelassenes Medikament hinreichend ersetzen können.	excellent - depends on person, alternative for drugs
OLP	Es ist ein komisches Gefühl, weil man sich zum einen wie **selbst "belügt**" und zum anderen hat man eine Erwartung, ob sie wirklich funktionieren wird. Doch die **Regelmässigkeit** (Einnahme) mit **Glaube** (Affirmationen) kombiniert, kann meiner Meinung nach einiges mit uns machen.	fair - mixed feelings, mindfulness
OLP	Ich hatte tatsächlich das Gefühl, dass es mir irgendwie **geholfen** hat, daher fand ich es **gut**.	good -
OLP	Finde ich **grundsätzlich gut**, jedoch würde ich es nicht weiterführen, da sie mir trotz all meiner Gedankenkraft und regelmässiger Einnahme für die Prüfung **nichts gebracht** haben.	good - didn't work
OLP	Grundsätzlich eine **sehr gute** Idee, wenn es bei mir (stärker) **genützt hätte**.	excellent - didn't work
OLP	Ich finde die Idee **sehr gut** und denke, dass darin ein großes **Potential** für die Zukunft der Psychologie/ Medizin steckt	excellent - potential
OLP	Finde ich **gut**.	good -
OLP	Ich glaube ehrlich gesagt **nicht wirklich** daran, dass diese offene Art funktioniert. Wenn es **verdeckt wäre jedoch schon**. Es war eher immer wieder ein kleiner Mut-Macher der mich an die positiven Gefühle erinnerte, zb dass ich auf mich Vertrauen kann	poor - deception would be better
OLP	Für Prüfungsangst sicher eine **gute** Idee... ein gutes Mittel um sich zu beruhigen	good - could help with certan things
OLP	Finde ich **in Ordnung**, wenn es hilft.	fair -
OLP	Ich bin mir **nicht sicher**. Ich hatte **nicht** das Gefühl, als würde mir die Pille speziell helfen, weniger Angst zu haben.	poor - didn't help
OLP	Ansich eine **gute Idee**, aber wahrscheinlich wirkt es **nicht bei jeder Person**.	good - depends on person
OLP	**Gleichgültig**.	fair -
IP	Ich finde es eine **gute Idee**, da man dann ohne wirklich etwas zu nehmen (Medikament/Beruhigungsmittel) eine Möglichkeit hat, die Symptome zu verringern.	good - decrease symptoms without active ingredients
IP	Ich finde es eine **sehr gute Idee** und habe vor es ab jetzt immer zu machen während meinen Prüfungsphasen. Ich war **noch nie so gut drauf während einer Prüfungsphase** vorallem war ich eifach generell weniger gestresst.	excellent - felt great
IP	Ich finde es eine **gute Idee**. Man muss keine künstlichen Wirkstoffe einnehmen und spart Geld.	good - decrease symptoms without active ingredients
IP	**gute Idee**, da keine Nebenwirkungen.	good - no side effects
IP	Am Anfang war es sehr **gewöhnungsbedürftig**, jedoch viel es mit der Zeit in meinen normalen Tagesrythmus und von dem her wurde es ganz normal für mich.	fair - needs getting used to (positive)

IP	**Gute Idee**, weil wir an regelmäßige Medikamenteneinnahmen oft gewöhnt sind, heisst man hat zweimal am Tag einen Moment der "Bewusstmachung", während dessen man sich auf eine positive Einstellung/Haltung/Effekt konzentriert und "runterkommt" versus einer sonst negativen und gestressten Haltung gegenüber einer Prüfung (oder auch anderen negativ besetzten Ereignissen).	good - mindfulness
IP	Ich finde es **gut**, dass man sich durch das einnehmen der Pille zweimal täglich mit der Prüfung auseinandersetzen muss. Ich hatte das Gefühl, dass die **Routine** meine Gedanken der Prüfung gegenüber ruhiger und geordneter wurden. Aber dass das genau durch die Form einer imaginären Pille geschehen musste fand ich auch ein wenig **eingrenzend**.	good - mindfulness
IP	Mir persönlich würde es sehr helfen eine echte Placebopille einzunehmen. Es würde sich **echter** anfühlen.	poor - need real placebo pill
IP	Ich finde es eine **gute** Idee. Dadurch kann man ein Erwünschtest Effekt "selbstständig" erzeugen **ohne pharmakologische** Mitteln einzunehmen die vielleicht **unerwünschte Nebenwirkungen** haben konnten	good - decrease symptoms without active ingredients, no side effects
IP	Ich finde es **sehr komisch**. Ich kann nicht viel dazu sagen.	poor - strange
IP	grundsätzlich **nicht schlecht**, die einnahme von "**richtigem**" placebo hätte mir aber mehr geholfen, den Effekt zu spüren	fair - need real placebo pill
IP	Ich finde das Experiment und die Idee spannend. Ich glaube, dass eine imaginäre Pille **wirken kann**. Meiner Meinung kommt es aber stark darauf an, **für was sie wirken soll** und wie stark die Not / das Leiden ist. Viele Faktoren könnten eine Rolle spielen / die Wirkung moderieren. Allerdings fand ich die Erfahrung interessant und würde es vielleicht selbst nochmal ausprobieren bei Bedarf.	fair - interesting
IP	Finde ich soweit eine **gute Idee**	good -
IP	**Gute Idee**, jedoch braucht es eine gewisse Offenheit. Mehr **Disziplin** es wirklich zu machen (braucht Konzentration ergo mehr Zeit und Kraft als physische Pille)	good - requires discipline
IP	Ich glaube, es **funktioniert nicht**. Da muss man andere Methoden finden.	poor - need find other methods
IP	Grundsätzlich halte ich es für eine **gute Idee**. Ich persönlich bin aber kein grosser Fan der Pilleneinnahme, weswegen es mich manchmal **gestresst** hat, jeden Tag an die Pille denken zu müssen, auch wenn ich mir eine Erinnerung gesetzt habe. Vermutlich würde sich dies jedoch ändern, wenn ich die Pille für einen längeren Zeitraum einnehmen würde und ich mich somit an die Einname der Pille gewöhnen würde.	good - required discipline too stressful, needs getting used to (positive)
IP	Sie gerät zu leicht in **Vergessenheit**, die Vorstellung. bleibt **zu abstrakt**, zu wenig physisch erfahrbar.	poor - too abstract, needs getting used to so you won't forget
IP	ich finde es **gut**, da man **mehr Zeit investiert**/ sich mehr mit der "Behandlung" **befasst**, da man ja den ganze Prozess der Pille (Einnahme -> Auflösung der Pille im Körper) sich vorstellen muss. und bei einer echten Pille, nimmt man die Pille ein und man wartet anschliessend auf die Wirkung der Pille.	good - mindfulness
IP	Grundsätzlich finde ich es eine spannende und auch **gute Idee**, doch durch meine Erfahrungen in dieser Studie, habe ich jetzt **nicht eine noch positivere** Einstellung gegenüber imaginären Pillen (aber a**uch nicht unbedingt eine schlechtere**).	good - no change in attitude
IP	Ich finde es auf jeden Fall **besser als eine "echte" Pille** einzunehmen und denke es kann nie schaden, ein wenig an Wunder zu glauben:)	fair - better than active ingredients
IP	ist eine **gute** Möglichkeit	good -
IP	Ich finde es grundsätzlich eine **gute** Sache (wenn es hilft)	good -
IP	**Sehr gute** Idee	excellent -
IP	Es **schadet sicher nicht**. Ein Versuch ist das wert	fair - won't hurt to try
IP	Wenn man wirklich daran **glaubt**, finde ich die Idee **sehr gut**. Ich hatte Zweifel daher glaubte ich auch nicht zu 100% daran.	excellent - only if you believe it, if you're not sceptical
IP	Ich finde die Idee grundsätzlich **sehr gut**. Allerdings hat sich bei mir nach ein paar Tagen ein **Gewöhnungseffekt** eingestellt, wodurch es mir schwerer viel, mir die Pille und ihre positiven Wirkungen erfolgreich vorzustellen. Vielleicht wäre eine Art "Refresher" nach einer gewissen Zeit sinnvoll.	excellent - smaller effect when getting used to
IP	Finde ich **gut**.	good -
IP	ich finde diese idee **interessant**, finde sie sollte weiter erforscht werden, weil es ohne viel aufwand **viele vorteile bringen könnte**	good - has potential
IP	Hat **nichts** gebracht :/	poor - didn't help
IP	Ich denke ich konnte es mir gut vorstellen, weil ich auch sonst immer Vitaminpräparate einnehme und weiss wie sich diese anfühlen. Ob das bei jemanden funktioniert der nicht so sensibilisiert ist, weiss ich nicht.	good - requires strong visualization abilities
IP	Ich finde es **besser** als richtige Pillen zu sich zu nehmen!	fair - better than active ingredients
IP	Sehr **interessanter** Ansatz und vielfältig einsetzbar	fair - interesting
IP	Ich denke die Idee ist **sehr gut**, wenn man ein gutes **Vorstellungsvermögen** hat. Ansonsten denke ich nicht dass es grosse Veränderungen gibt, ausser man steigert sich intensiver hinein.	excellent - requires strong visualization abilities
IP	Ich bin von dieser Idee **nicht** wirklich überzeugt.	poor - not convinced
IP	Ich finde es eine **gute** Idee	good -

IP	Finde ich eine **gute** Idee, aber das **Problem des vergessen** ist dann noch grössere bei mir	good - requires discipline, needs getting used to sp you won't forget
IP	Ich finde es grundsätzlich eine **gute** Idee, aber evtl. in einem anderen Setting. Für mich ist Prüfungsangst ein zu diffuses Phänomen, das durch sehr viele andere Faktoren auch beeinflusst werden kann. Ich denke mir würde eine imaginäre Pille bei zeitnaheren, **spezifischeren** Beschwerden **besser** helfen.	good - needs to be more specific
IP	Sie ist gut, der Ablauf muss aber **geübt** werden	good - needs getting used to
IP	Die Idee finde ich **spannend**.	good - interesting
IP	würde ich vermutlich **nicht** nochmal machen, da ich **keine** Wirkung verspürte	bad - didn't help
	**Nicht** viel, weil es kaum etwas gebracht hat.	
IP	Die Prozedur des „Einnehmens" verursachte bei mir **Wut**.	poor - didn't help
IP	**Gute** Idee, würde ich gerne weiterhin anwenden.	good -
IP	Ich finde die Idee **gut**, nur hat es bei mir nicht geholfen. Dies liegt aber daran, dass ich **mental nicht sehr stark bin** und dies auch im Leistungssport meine Schwäche ist.	good - requires strong visualization abilities
IP	Ich denke, das es eine **gute** Idee ist. Es ist toll, sich selbst zu helfen, **ohne echte Pillen** zu nehmen	good - decrease symptoms without active ingredients
IP	Falls diese Wirkung zeigen sollte über die Studie hinweg, dann ist es meiner Meinung nach eine **bessere Option als eine Pharmakologische** Pille zu nehmen.	fair - decrease symptoms without active ingredients
IP	im vorhinein war ich skeptisch, jetzt muss ich sagen: **genial**!	excellent -
IP	Finde ich eine **gute** Idee, wenn man sich total darauf **einlassen kann** & auch daran **glaubt**.	good - only if you believe it
IP	falls es funktioniert für Patient*innen, ist es eine **sehr gute** Idee	excellent -
IP	Wie gesagt, mir **fehlt** bei der Einnahme die **Haptik**.	poor - need real/ tangible pill
IP	Ich denke es kommt sehr auf die Person drauf an. Und ich denke es **muss nicht unbedingt eine Pille sein**. Vielleicht ein Schnipsen oder so. Dann fühlt man sich weniger "**verarscht**" und fokussiert sich auf den Kern der Behandlung - Symptome zu "kontrollieren". Aber eben, manche kontrollieren und verstehen gerne und andere nehmen gerne eine Pille.	fair - depends on person, doesn't need to be a pill
IP	**Gut** eigentlich.	fair -
IP	Wenn auch mit der imaginären Pille Wirkungen erzielt werden können, dann könnte ich mir vorstellen, dass es einigen Menschen starke Stresssituationen **etwas erleichtern könnte**. Ähnlich wie auch Meditation, Atemtechniken, an etwas Schönes/Gutes denken, etc.	fair - mindfulness
IP	Finde ich eine **gute** Sache, man kann sich besser auf sich selber konzentrieren und sich mit dem Stress abfinden.	good - mindfulness
IP	Ich finde es eine **sinnvolle** Idee.	good -
IP	Ich bin eher skeptisch wie bereits beschrieben denke ich das es eine **externe und materielle Quelle braucht**, auf die sich der Glaube fokussieren kann.	poor - need real/ tangible pill

**Table S8.**	**Credibility of explanation.** How credible did you find the explanation of why an imaginary pill / open label placebo can work?	**Helpfulness of explanation.** How helpful did you find the explanation of why the imaginary pill / open label placebo can work?
**group**	**credibility**	**helpfulness**
OLP	stark	mässig
OLP	mässig	mässig
OLP	stark	stark
OLP	extrem	extrem
OLP	stark	mässig
OLP	mässig	mässig
OLP	stark	stark
OLP	extrem	extrem
OLP	stark	stark
OLP	stark	mässig
OLP	stark	stark
OLP	stark	stark
OLP	stark	mässig
OLP	stark	stark
OLP	extrem	stark
OLP	stark	extrem
OLP	mässig	stark
OLP	mässig	mässig
OLP	stark	stark
OLP	mässig	stark

OLP	stark	stark
OLP	stark	stark
OLP	extrem	extrem
OLP	stark	mässig
OLP	stark	mässig
OLP	stark	mässig
OLP	mässig	mässig
OLP	stark	mässig
OLP	stark	extrem
OLP	minimal	extrem
OLP	stark	stark
OLP	mässig	extrem
OLP	extrem	extrem
OLP	extrem	stark
OLP	mässig	mässig
OLP	stark	stark
OLP	stark	stark
OLP	stark	mässig
OLP	stark	stark
OLP	stark	stark
OLP	kaum	mässig
OLP	stark	stark
OLP	stark	stark
OLP	mässig	mässig
OLP	mässig	mässig
OLP	mässig	mässig
OLP	extrem	extrem
OLP	stark	stark
OLP	stark	stark
OLP	stark	stark
OLP	mässig	mässig
OLP	stark	stark
OLP	stark	stark
OLP	mässig	mässig
OLP	mässig	stark
OLP	stark	stark
OLP	mässig	mässig
OLP	stark	stark
OLP	minimal	stark
IP	stark	stark
IP	mässig	stark
IP	stark	mässig
IP	stark	stark
IP	mässig	stark
IP	mässig	mässig
IP	mässig	mässig
IP	stark	stark
IP	mässig	stark
IP	minimal	minimal
IP	mässig	stark
IP	mässig	stark
IP	stark	stark
IP	stark	stark
IP	mässig	kaum
IP	stark	stark
IP	mässig	mässig
IP	stark	stark
IP	stark	stark
IP	extrem	stark
IP	mässig	mässig
IP	stark	stark
IP	stark	stark
IP	stark	stark
IP	stark	mässig

IP	stark	stark
IP	stark	stark
IP	mässig	mässig
IP	mässig	stark
IP	stark	stark
IP	mässig	stark
IP	extrem	extrem
IP	mässig	mässig
IP	minimal	minimal
IP	stark	stark
IP	stark	stark
IP	stark	mässig
IP	mässig	stark
IP	stark	mässig
IP	stark	mässig
IP	überhaupt nicht	überhaupt nicht
IP	stark	mässig
IP	stark	stark
IP	mässig	mässig
IP	stark	stark
IP	stark	stark
IP	mässig	mässig
IP	extrem	extrem
IP	kaum	kaum
IP	stark	stark
IP	mässig	stark
IP	stark	mässig
IP	stark	stark
IP	stark	stark
IP	mässig	stark

S9 Table. Learning during treatment. Did you learn anything from participating in this treatment study? If yes, what?

group	learned OLP_IP (Haben Sie durch die Teilnahme an dieser Behandlungsstudie etwas gelernt? Wenn ja, was?)	learnings
OLP	Ich habe mehr über die Wirkmechanismen der Placebopille erfahren.	more about placebo mechanism
OLP	dass es schwieriger ist regelmässig die pillen einzunehmen mit dem gedanke dass es placebo ist	that adherence is difficult
OLP	Fällt mir nichts ein	N/A
OLP	Etwas über mich selbst	something about myself
OLP	Es hat mir geholfen, mich frühzeitig mit der Prüfungsangst auseinanderzusetzen. Ich habe das Gefühl, dass man die Angst dann in kleineren Portionen aufteilt. Und eventuell einen grösseren Angstanfall vermeidet.	how to deal with anxiety
OLP	Diese Prüfungssituation war sehr anders, als normale Prüfungssituationen bei mir. Durch Corona konnte ich alles von zu Hause aus und online machen.	N/A
OLP	selbstaufmerksamkeit ist wichtig	that mindfulness can help a lot
OLP	nein	no
OLP	habe den positiven Aspekt der Placebo-Wirkung erfahren	how powerful our psyche/ imagination can be
OLP	Nein eigentlich nicht direkt. Ich habe bisher mit Homöopathie gearbeitet, kenne also die Art der "Wirkung" bereits.	no
OLP	Body knows and senses a lot more than what you may be aware of	that mindfulness can be very helpful
OLP	Nein	no
OLP	Mich immer wieder an gute Dinge zu erinnern kann helfen	have more self confidence
OLP	Interessanterweise habe ich gedacht, dass Placebos (offen) auch wirken, ohne, dass ich daran denken muss (aktiv). Also ich war skeptisch, habe gedacht ich müsste nicht daran glauben, dass sie wirkt, bekomme dann gesagt, dass ich dennoch 2-Mal täglich an das Gefühl denken soll, wie ich die Prüfung lösen will. War ein kleiner Widerspruch. [Codierung bezieht sich auf Scepticism-Frage]	no
OLP	Dass ich mir normalerweise viel zu viele Sorgen mache und Prüfungen entspannter entgegentreten sollte	have more self confidence

OLP	Ja, mehr über Placebos und deren Effekt. Ich hätte nicht gedacht, dass sie auch eine Wirkung haben wenn man bewusst weiss, dass es keinen Wirkstoff darin hat.	more about placebo mechanism
OLP	Ich habe gelernt, dass mir eine Routine (hier die Pilleneinnahme) bei meiner Angst helfen kann. Ich denke aber, dass meine Angst vor allem durch mein Mindset beeinflusst wird und weniger von der aktiven Einnahme einer Placebopille.	how to deal with anxiety
OLP	Nein	no
OLP	Ich kann jetzt nachvollziehen, dass Placebo Interventionen auch gut funktionieren können.	that OLPs can actually work
OLP	eigentlich habe ich nur gelernt, dass placebos auch wirken können, wenn man weiß, dass es placebos sind	that OLPs can actually work
OLP	Dass es anscheinend tatsächlich wirkt, oder ich habe mir einfach gewünscht dass es wirkt	that OLPs can actually work
OLP	Mehr auf mich selbst zu vertrauen. Die Placebo Pille hat mir mit der Einnahme auch dabei geholfen einmal am Tag meine Gedanken zu sammeln und mir zu sagen, dass ich das schaffe	have more self confidence
OLP	Erwartungen sind wichtig	how important expectations are
OLP	Dass offen verabreichte Placebopillen für wirksam gehalten werden.	more about placebo mechanism
OLP	-	N/A
OLP	Das offene placebos funktionieren können	that OLPs can actually work
OLP	Psychologische Phänomene haben eine starke biologische Basis	more about placebo mechanism
OLP	Dass, wenn man sich an einen Plan hält, Tabletten schluckt, dass die Psyche manipulieren kann, obwohl man weiss, dass die Tabletten selber nichts ändern, sondern nur, die Psyche, die sich an den Plan hält.	that daily routines help clearing thoughts, that mindfulness can be very helpful
OLP	Das Placebopillen mir helfen können, mich weniger ängstlich zu fühlen.	how to deal with anxiety
OLP	Nein	no
OLP	Das die Placebobehandlung mir subjektiv weniger geholfen hat als ich dachte, aber dass ich immer noch denke, dass es eine Wirkung geben kann.	that OLPs can actually work
OLP	Nein	no
OLP	Das die Kraft des Denkens grösser ist, als ich erwartet habe	how powerful our psyche/ imagination can be
OLP	Dass Placebo besser wirkt als ich gedacht habe.	how powerful our psyche/ imagination can be
OLP	Nein	no
OLP	Nein	no
OLP	Placebos wirken (für mich) vor allem bei körperlichen Symptomen.	more about placebo mechanism
OLP	Zu sehen wie es meinem Körper vor der Prüfung geht	that mindfulness can be very helpful
OLP	Ja, dass sich alles in unserem Kopf abspielt und wir mit dem richtigen Mindset alles schaffen können.	how powerful our psyche/ imagination can be
OLP	Das man sich Momente der Ruhe nehmen sollte, auch in stressigen Zeiten.	that mindfulness can be very helpful
OLP	Routine hilft bei der Prüfungsvorbereitung. Sei es die Einnahme von Placebotabletten oder das Lutschen von Bonbons. Das verleiht Sicherheit und Zuversicht.	that daily routines help clearing thoughts, have more self confidence
OLP	Es gibt Versuche mit offener Placebo-Abgabe.	more about placebo mechanism
OLP	Ich denke eins der wichtigsten Dinge, die ich in dieser Lernphase durch die Placebopillen gelernt habe, ist, dass ein Moment der Ruhe, des Durchatmens sehr viel bringen kann. Einen Moment bewusst darauf zu richten, dass alles schon irgendwie klappen wird und dass ich das alles schaffen werde, das verändert manchmal die ganze Stimmung, die man im Augenblick zuvor hatte. Und damit auch die Stimmung der ganzen Lernphase...	how important expectations are, that mindfulness can be very helpful
OLP	Regelmässige, Zeitgerechte Pilleneinnahme schwerer als gedacht	that adherence is difficult
OLP	Dass es in manchen Situation hilft, wenn man seine mentale Befindlichkeit probiert in Griff zu kriegen mit Täuschungen, sei es in Form von Placebopillen oder einfache Sätze, die man sich selbst einredet.	that mindfulness can be very helpful
OLP	Ja, Verstand spielt eine wichtige Rolle, ich war überzeugt (und begeistert) von die Auswirkung von Placebo, aber ich denke wenn ich nicht gewusst hätte dass diese Pille Placebo wären, hätte ich mehrere Verbesserungen. Aber die Studie war sehr spannend!	how powerful our psyche/ imagination can be, that OLPs can work
OLP	nein, für mich war nichts Neues dabei	no
OLP	Dass tägliche Affirmation etwas bewirkt hat. Ich war an der Prüfung nicht hektisch und war ziemlich gelassen.	that mindfulness can be very helpful
OLP	Mentale Einstellung macht einiges aus ;)	how powerful our psyche/ imagination can be
OLP	Ja, regelmässige Einnahme von Tabletten. Z.T. auch dass Gedanken helfen können.	that adherence is difficult, how powerful our psyche/ imagination can be
OLP	Ich hatte dadurch etwas mehr Hoffnung für mein Abschneiden, da ich auf die Wirkung der Pille vertraut habe.	how important expectations are
OLP	Dass aktuell zu Placebos geforscht wird.	more about placebo mechanism
OLP	Ja, ich habe gelernt, wie nur der Glaube an eine Placebopille eine Wirkung zeigen kann.	how powerful our psyche/ imagination can be

OLP	Dass man anscheinend auch durch offene Verabreichung von Placebos Wirkung erzeugen kann, davon bin ich aber nicht ganz überzeugt	that OLPs can actually work
OLP	Ja aber mehr für mich persönlich, dass man sich an manchen Dingen „festhalten“ kann und sich immer wieder erinnern kann dass man gut vorbereitet ist und dass man etwas schaffen kann.	have more self confidence, that mindfulness can be very helpful
OLP	Nein	no
OLP	Es gibt warscheinlich diverse Wege weniger Prüfungsangst zu haben. Ich werde mich auf jedenfall im nächsten Semester darsuf achten, wie es ohne Pille ist.	how to deal with anxiety
OLP	Dass es nützlich sein kann sich einen moment Zeit zu nehmen & daran zu denken, dass man aufmerksam sein soll / kann.	that mindfulness can be very helpful
OLP	Ich habe gelernt das trotz des Wissens, das man eine Placebopille zu sich nimmt, eine Wirkung stattfinden kann.	that OLPs can actually work
IP	Ich habe gelernt in stressigen Momenten mich auf ein gutes Gefühl zu konzentrierten/erinnern und mich dadurch ein Stück weit zu beruhigen.	that mindfulness can be very helpful, how to deal with anxiety
IP	Es kann helfen, wenn man eifach zwei Mal am Tag daran denkt, diese imaginäre Pille zu nehmen. Ich weiss nicht was es war, aber es gab mir schon mehr Sicherheit. Ich hatte nicht einmal einen Moment an dem ich so gestresst war, dass ich anhalten musste und mich sammeln, sonst hatte ich dieses Gefühl.	that mindfulness can be very helpful
IP	Es ist möglich sehr viel nur mit den Gedanken und dem Glauben, dass etwas wirkt, zu erreichen	how powerful our psyche/ imagination can be
IP	-	N/A
IP	wie aufwändig so eine Studie sein kann	that adherence is difficult
IP	Es hilft, kontinuierlich (täglich) mit den eigenen Gedanken, Gefühlen und körperlichen Auswirkungen zu arbeiten, wenn man versuchen will gegebüber einer Situation eine andere Haltung einzunehmen. Wenn man sich 2x täglich auf einen bestimmten greifbaren Effekt (auch physisch!) konzentriert, ein Problem bewusst macht und explizit mit einer positiven Handlung und Haltung ersetzt, dann hilft das.	that daily routines help to clear thoughts, that mindfulness can be very helpful
IP	Das mich Routine dazu bringt, meine Gedanken zu ordnen. Durch dass ich täglich an das selbe denken musste, habe ich auch mich selbst besser kennengelernt und wusste plötzlich was mich wirklich genau an einer Prüfung zum stressen bringt.	something about myself, that daily routines help to clear thoughts
IP	Das es möglich ist, eine positiven Effekt zu spüren, aber vielleicht in Zusammenhang mit einem vorherigen Training dazu.	that IPs can actually work and be used daily
IP	Jeden Tag versuchen entspannt zu sein, hilft im Alltag weniger nervös zu sein	that mindfulness can be very helpful
IP	Nein, ich habe dazu nicht neues gelernt.	no
IP	dass der Placebo Effekt schon bei der alleinigen Vorstellung der Einnahme ausgelöst werden kann	how powerful our psyche/ imagination can be
IP	Ja ich habe eine potentielle neue Therapiemöglichkeit gelernt. Veränderung allein durch die Vorstellungskraft. ich finde das sehr spannend und freue mich, die Ergebnisse der Studie zu erfahren.	about a potentially new therapy method, how powerful our psyche/ imagination can be
IP	Nein	no
IP	Viel über Placebo nachgedacht und herkömmliche Behandlungen kritisch hinterfragt	to question conventional methods
IP	nein	no
IP	Ich habe gelernt, dass es sehr wichtig ist, an sich selbst zu glauben. Ich bin davon überzeugt, dass man sich auch selbst Mut zusprechen kann und dies auch als eine Art imaginäre Pille wirken könnte.	have more self confidence
IP	Innehalten, kurze Übungen regelmässig in Alltag einbauen	that mindfulness can be very helpful
IP	ich habe für mich gelernt, dass wenn ich ca. 3 min am Tag opfere, mich kurz "beruhigen" lasse (in diesem Fall durch die Wirkung der imaginären Pille), lässt sich auch meine Nervosität reduzieren.	that daily routines help to clear thoughts
IP	wie wichtig es ist, an die Wirkung (egal ob imaginäre Pille oder Open-label Placebo) zu glauben	how important expectations are
IP	Dass die Gedanken eine enorme Macht haben.	how powerful our psyche/ imagination can be
IP	-	N/A
IP	Nein	no
IP	das man sich Sachen eigentlich sehr leicht einreden kann, mit der richtigen Überzeugung	how powerful our psyche/ imagination can be
IP	Nein	no
IP	Meine Selbstsicherheit wurde gestärkt. Ich denke wenn ich mehr an mich selber glauben würde, dann bräuchte ich auch nicht mal die imaginäre Pille. Sehr wahrscheinlich war ich vor allem skeptisch der Pille gegenüber, wenn ich bei der Einnahme der Pille nicht an mich geglaubt habe und somit erhoffte, meine Gefühle würden sich durch diese imaginäre Pille ändern, tat es aber jedoch nicht.	have more self confidence

IP	Ja, dass man durch die Vorstellung Gedanken, Emotionen und Körperempfindungen beeinflussen kann.	how powerful our psyche/ imagination can be
IP	Die Wichtigkeit & Wirkungskraft der Imagination	how powerful our psyche/ imagination can be
IP	-	N/A
IP	Man kann sich alles vorstellen und sich das jeden tag wie mantras vorsagen	that daily routines help to clear thoughts, about the power of our psyche/ imagination
IP	Mehr Momente innehalten und im Moment sein, den Fokus zurück erlangen und selbstberuhigende Strategie	that mindfulness can be very helpful, that IPs can actually work and be used daily
IP	Dass man wirklich mit Placebo-Pillen Krankheiten oder Schmerzen lindern kann. Ich habe bereits vom Placebo-Effekt gehört, wusste aber nicht, dass man das bereits offen den Patienten verabreicht.	about a potentially new therapy method
IP	Ich werde dieses Vorgehen auch für andere Bereiche für mich selbst einsetzen	how to deal with anxiety, that IPs can actually work and be used daily
IP	Ja, ich wusste nicht dass es möglich sein könnte mit gutem Vorstellungsvermögen wirklich weniger stress haben zu können.	how powerful our psyche/ imagination can be
IP	Leider nicht wirklich.	no
IP	Wie einflussreich die Psyche sein kann:)	how powerful our psyche/ imagination can be
IP	nein	no
IP	Dass man sich selber gut beruhigen, ermutigen kann.	how powerful our psyche/ imagination can be
IP	Ja, Placebos haben eine starke Wirkung in verschiedenen Formen	that IPs can actually work and be used daily
IP	Vorstellen erfordert eine Anstrengung. Angeleitet während des Briefings war dies viel einfacher zu bewältigen, als im Alltag.	that adherence is difficult
IP	nein	no
IP	Dass ich während der Lernzeit (unter Stress) sehr skeptisch bin gegenüber Neuem und gegenüber Dingen, die mir Zeit/Energie rauben (wenn auch nur sehr wenig).	something about myself
IP	Immer offen zu sein für neue Dinge, ich werde die imaginäre Pille auch in der nächsten Prüfungsphase anwenden.	to always be open minded towards new things, that IPs can actually work and be used daily, how to deal with anxiety
IP	Dass ich etwas gegen meine Prüfungsangst unternehmen muss.	how to deal with anxiety
IP	Nein	no
IP	Selbst-Manipulation (via imaginärer Pille) ist ein relevanter Schritt zur erfolgreichen Selbstregulation und dient als Coping-Möglichkeit in Bezug auf Stress.	how to deal with anxiety, that IPs can actually work and be used daily
IP	Ich werde die imaginäre Pille vermutlich in weiteren schwierigen Situationen einsetzten, wenn ich den Bedarf verspüre.	that IPs can actually work and be used daily
IP	Mehr Selbstvertrauen in mich zu haben	have more self confidence
IP	nein	no
IP	Nein.	no
IP	JA! Vor allem durch das Zoommeeting. Dass man sich an Situationen erinnern kann, wo man sich "gut fühlte" und man sich dieses Gefühl dann vorstellt in schwierigen Situationen.	about the power of our psyche/ imagination
IP	Dass man auch mal an seine eigenen Leistungen glauben kann und dass viele Dinge im Leben Kopfsache sind.	have more self confidence
IP	Ich kann mir vorstellen, die imaginäre Pille in weiteren Stresssituationen einzunehmen, wodurch ich mich mit den positiven Effekte der Pille beschäftigen kann, sodass ich mich bei starker Aufregung/Nervosität etwas beruhigen kann.	how to deal with anxiety, that IPs can actually work and be used daily
IP	Ich habe gelernt wie ich mich besser auf mich konzentrieren kann.	that mindfulness can be very helpful
IP	Ja mich mit einer imaginären Pille zu entspannen.	that IPs can actually work and be used daily
IP	Nicht wirklich.	no
